# Synthetic mismatches enable specific CRISPR-Cas12a-based detection of genome-wide SNVs tracked by ARTEMIS

**DOI:** 10.1016/j.crmeth.2024.100912

**Published:** 2024-12-06

**Authors:** Kavish A.V. Kohabir, Jasper Linthorst, Lars O. Nooi, Rick Brouwer, Rob M.F. Wolthuis, Erik A. Sistermans

**Affiliations:** 1 Department of Human Genetics, Amsterdam UMC location Vrije Universiteit Amsterdam, Amsterdam, the Netherlands; 2 Amsterdam Reproduction & Development, Amsterdam, the Netherlands; 3 Imaging and Biomarkers, Cancer Center Amsterdam, Amsterdam, the Netherlands; 4 Amsterdam Institute for Immunology and Infectious Diseases, Amsterdam, the Netherlands; 5 Clinical Laboratory, Unilabs, Enschede, the Netherlands; 6 Cancer Biology and Immunology, Cancer Center Amsterdam, Amsterdam, the Netherlands

**Keywords:** CRISPR, Cas12a, SNP, SNV, diagnostics, cancer, BRAF V600E, melanoma, liquid biopsy, cfDNA

## Abstract

Detection of pathogenic DNA variants is vital in cancer diagnostics and treatment monitoring. While CRISPR-based diagnostics (CRISPRdx) offer promising avenues for cost-effective, rapid, and point-of-care testing, achieving single-nucleotide detection fidelity remains challenging. We present an *in silico* pipeline that scans the human genome for targeting pathogenic mutations in the seed region (ARTEMIS), the most stringent crRNA domain. ARTEMIS identified 12% of pathogenic SNVs as Cas12a recognizable, including 928 cancer-associated variants such as *BRAF*^*V600E*^, *BRCA2*^*E1953∗*^, *TP53*^*V272M*^, and *ALDH2*^*E504K*^. Cas12a exhibited remarkable tolerance to single mismatches within the seed region. Introducing deliberate synthetic mismatches within the seed region yielded on-target activity with single-nucleotide fidelity. Both positioning and nucleobase types of mismatches influenced detection accuracy. With improved specificity, Cas12a could accurately detect and semi-quantify *BRAF*^*V600E*^ in cfDNA from cell lines and patient liquid biopsies. These results provide insights toward rationalized crRNA design for high-fidelity CRISPRdx, supporting personalized and cost-efficient healthcare solutions in oncologic diagnostics.

## Highlights

• ARTEMIS finds genome-wide SNVs in Cas12a seed regions for CRISPR diagnostics
• Cas12a tolerates single-nucleotide mismatches, even within its seed region
• Synthetic mismatches in the crRNA yield single-nucleotide detection specificity
• This strategy works on various sample types and different clinically relevant SNVs

## Motivation

While CRISPR-based diagnostics (CRISPRdx) offer promising avenues for cost-effective, rapid, and reliable testing, achieving single-nucleotide detection fidelity remains challenging. We previously demonstrated that optimization, based on Cas12a variant choice and titration of target amplification, often came at the expense of assay speed. Since this optimization focused exclusively on a specific set of SNVs just beyond the seed region, we envisioned that optimizing other SNVs of interest would be tedious and empirical, failing to fully leverage the flexibility of CRISPRdx. For these reasons, we examined here whether rationalized crRNA design and mutagenesis could provide a more generalizable approach to single-nucleotide-specific CRISPRdx.

## Graphical abstract

**Figure:**
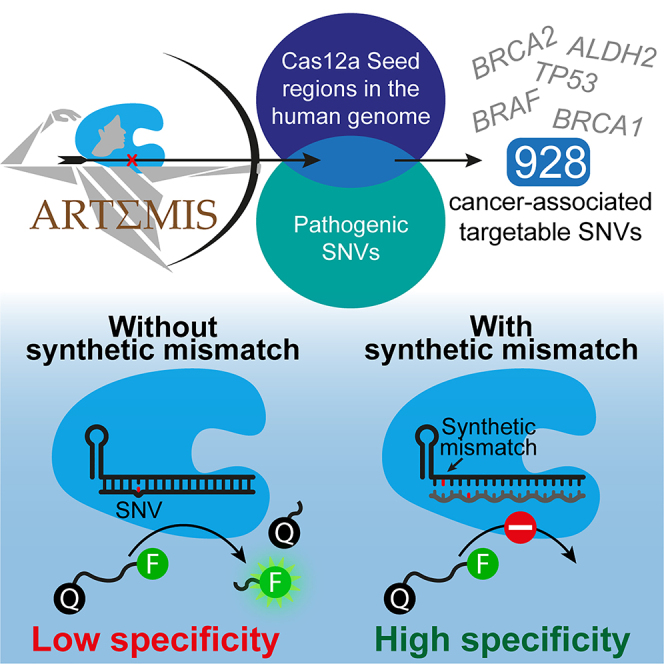

## Teaser

Cas12a-based SNV detection often yields false positives due to mismatch tolerance. Kohabir et al. present ARTEMIS, a pipeline pinpointing clinically relevant SNVs within Cas12a seed regions. By incorporating synthetic mismatches, their method enhances single-nucleotide fidelity in CRISPR-based detection of several cancer-associated variants, including *BRAF*^*V600E*^, promising precise and cost-effective genetic diagnostics.

## Introduction

Early detection of cancer onset or cancer relapse is important for adequate therapeutic intervention and improved chances of survival. Advances in liquid biopsy-based diagnostics based on genetic and fragmentomic biomarkers found in cell-free tumor-derived DNA (ctDNA) make the use of ctDNA as a biomarker for minimally invasive detection of malignancies feasible.^1^^,^^2^ However, achieving the necessary sensitivity and specificity for the non-invasive detection of cancer-associated SNVs remains challenging. Current methods are primarily reliant on next-generation sequencing (NGS) and often lack broad accessibility, cost-effectiveness, and user-friendliness, limiting their practicality in (point-of-care) clinical settings and resource-constrained areas. Addressing these challenges requires innovative approaches, such as leveraging the capabilities of CRISPR systems, which have revolutionized genomic engineering and hold promise for advancing cancer diagnostics.

For over a decade now, synthetic biology enabled bacterial RNA-guided endonucleases, associated with CRISPR, to be repurposed as programmable molecular tools for various genomic manipulations.^3^^,^^4^^,^^5^^,^^6^ In particular, class 2 systems, including CRISPR-Cas9, have been thoroughly characterized and adopted as genome editors since all required nucleolytic properties are catalyzed by a single protein. CRISPR-Cas12a (previously named CRISPR-Cpf1) was the first characterized type V system, a highly diversified CRISPR endonuclease family among class 2 systems.^7^ Many effector members of this family are RNA-guided endonucleases that upon protospacer-adjacent motif (PAM)-dependent recognition of an activator target sequence in *cis*, reveal indiscriminate collateral single-stranded DNase activity in *trans.* This target-dependent *trans*-nuclease property of type V nucleases can be repurposed for specific, fast, cost-efficient, multiplexable, and point-of-care CRISPR-based diagnostics (CRISPRdx).^8^

**Figure 1:**
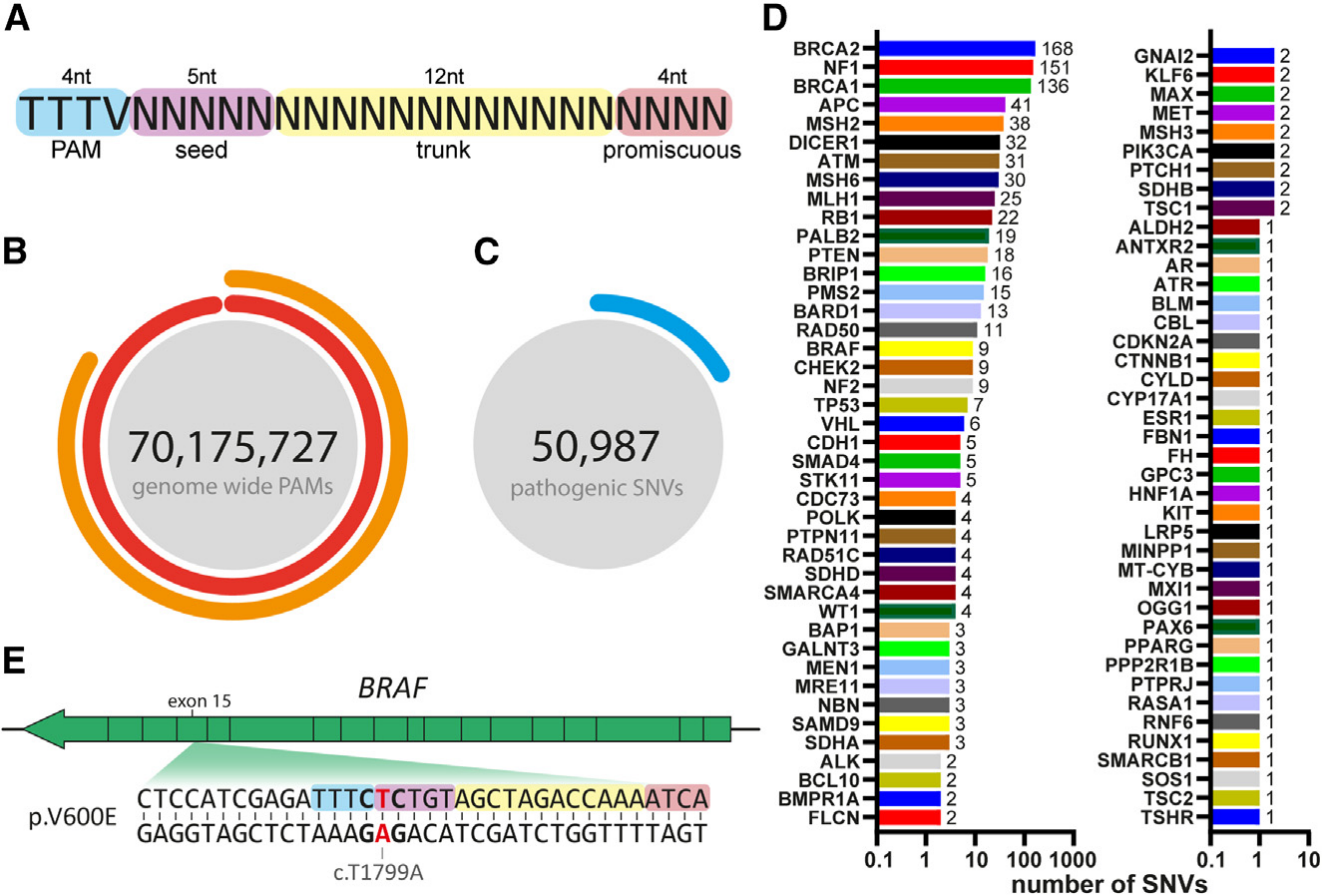
Outcomes of ARTEMIS for Cas12a-based detection of SNVs (A) A total of 21-nt Cas12a target sites are preceded by a 5′ tetranucleotide PAM sequence. The first five PAM-proximal nucleotides of the target site are complementary to the seed sequence of the crRNA. (B) ARTEMIS identifies how many PAM sequences are found genome-wide and what percentage of these are not coinciding with common SNVs (minor allele frequency > 1%) or have overlapping seed sequences. (C) ARTEMIS finds that of all found pathogenic SNVs, roughly 12% colocalize within the Cas12a seed sequence. (D) Amount of found cancer-associated SNVs within Cas12a seed regions per gene. An overview of targetable SNVs per chromosome can be found in Figure S1B. (E) A common oncogenic SNV found is BRAF c.T1799A, which is a missense mutation at PAM+1, resulting in protein variant p.V600E.

Type V CRISPR ribonucleoproteins interact with target DNA by PAM-dependent R-loop initiation, RNA-DNA heteroduplex formation, further strand displacement, and often, subsequent catalytic substrate hydrolysis.^9^^,^^10^ In this series of events, PAM recognition and strand invasion are most crucial to allow for sufficient binding affinity and succession toward target cleavage. Therefore, mismatches in the PAM or the adjacent antisense pentanucleotide stretch referred to as the seed region (Figure 1A) are usually worse tolerated, as they destabilize the complex and may abort the cleavage reaction.^9^^,^^10^ Previously, we demonstrated Cas12a-based detection of frequently occurring cancer-associated point mutations in *TP53* beyond the seed region.^11^ We showed that detection accuracy outside the seed region could be optimized by using non-engineered, physiological LbCas12a and by reducing the extent of target DNA amplification. This was often at the cost of assay speed, since reducing amplification lowered the number of activators, yielding slower assay kinetics and thus a prolonged assay duration.

Instead of tweaking Cas12a or activator properties, CRISPR RNA (crRNA) engineering may offer alternative approaches to achieve enhanced specificity. In this study, we explore the tolerance of Cas12a to deliberately introduced mismatches, termed synthetic mismatches (SMs), in the crRNA and their effect on SNV detection accuracy. Targeting mutations within the seed region and introducing SMs on top of that should theoretically achieve high-fidelity detection, given the anticipated stringency of the seed region.

This study evaluates the applicability of Cas12a for CRISPRdx of cancer-associated SNVs across the human genome. To facilitate finding clinically relevant SNVs located within Cas12a-targetable seed regions, we developed a searching algorithm for targeting pathogenic mutations in the seed region (ARTEMIS). Focusing on cancer-associated SNVs, we demonstrate that even within the seed region, Cas12a-based SNV detection is not specific per se, but that crRNA engineering of the seed region can yield high-fidelity detection of *BRAF* p.V600E on synthetic target oligonucleotides, cell culture-derived material, and liquid biopsy material. Our findings underscore the potential for rapid, cost-effective, and point-of-care cancer CRISPRdx with single-nucleotide precision, offering promising advancements in the field.

## Results

### ARTEMIS

We have previously shown that Cas12a-based diagnostics for point mutations beyond the seed region can be non-specific, requiring empirical tailored optimization to distinguish between the variant of interest and co-amplified wild-type (WT) material.^11^ Since mismatch tolerance is expected to be lowest in the seed region,^9^^,^^10^ proper assay design could take this into account by detecting the variant of interest within the crRNA seed region, theoretically yielding an improved diagnostic test accuracy.

We therefore developed a pipeline that explores the human genome and identifies loci for ARTEMIS by scanning for all occurring PAM instances within the human reference genome (GRCh38). Of all 70,175,727 found PAMs, 861,580 (∼1.2%) overlapped with common SNPs (allele frequency >1%), making them unsuitable for developing a broad range test, and were therefore excluded (Figure S1A). Furthermore, 10,558,576‬ (∼15%) PAMs belong to partially overlapping seed regions, allowing for multiple options to target a certain SNV of interest within a Cas12a seed region (Figure 1B). After merging the overlapping seed regions, we found a total of 59,617,151 distinctive areas within at least one Cas12a-targetable seed region, spanning 328,947,938 bp (∼10% of the human genome). Among all 50,987 human pathogenic SNVs, as annotated in ClinVar,^12^ we found that 6,243 (∼12%) are located within Cas12a seed regions (Figure 1C). Sub-filtering for cancer-associated SNVs leaves a list of 928 cancer-associated SNVs that can be targeted within the seed region using Cas12a (Data S1).

This list includes well-characterized commonly observed mutations in cancer-related genes such as *BRCA1*, *BRCA2*, *RB1*, *TP53*, and *BRAF* (Figure 1D)*.* As a proof of principle, we focused on *BRAF* p.V600E (c.T1799A; dbSNP: rs113488022), a gain-of-function hotspot mutation at PAM+1 commonly found in but not unique to melanoma, thyroid cancer, and colorectal cancer (Figure 1E).^13^ p.V600E is found in >90% of all *BRAF*-mutated tumors, making it an interesting candidate for targeted early-onset detection, treatment efficacy monitoring, or detection of relapses.^14^^,^^15^^,^^16^^,^^17^ Notably, the European Society of Medical Oncology and the National Comprehensive Cancer Network guidelines recommend mutational analysis of *BRAF*^*V600*^ to better guide treatment decisions,^18^^,^^19^ as the overactive mutant protein can be targeted therapeutically with BRAF inhibitors such as dabrafenib, vemurafenib, or encorafenib.^20^^,^^21^

### Cas12a is tolerant to single mismatches in the seed region

To visualize and quantify Cas12a collateral activity, we made use of a quenched fluorescent reporter assay that allowed us to monitor Cas12a *trans*-cleavage activity over time. Using KR105, a crRNA that recognizes the *BRAF* p.V600E variant, we verified that all reaction components (i.e., Cas12a, crRNA, activator, and probe) in a pH-buffered solution are required to catalyze an increase in fluorescence signal (Figures S2A and S2B). This was determined using all 16 possible combinations of these reaction components. Significant fluorescence signal increase above background was observed, which could be quantified by comparing the fluorescence curve slopes (Figure S2C).

**Figure 2:**
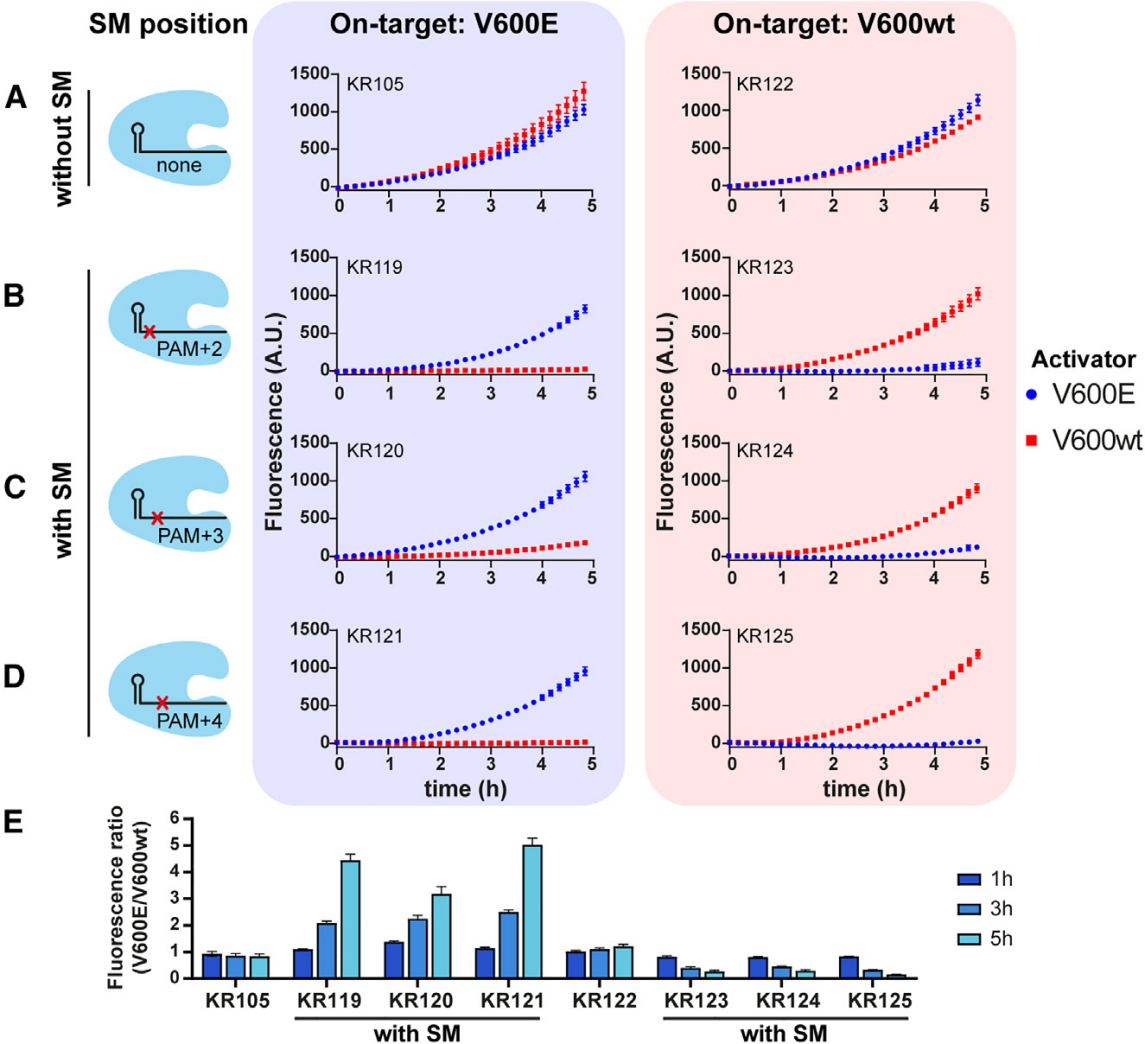
Introducing synthetic mismatches in the crRNA yields single-nucleotide fidelity BRAF p.V600E detection (A) crRNAs for p.V600E and wild-type (WT) BRAF detection (KR105 and KR122, respectively) were used in CRISPRdx reactions containing either V600E or WT activator. The variant of interest causing p.V600E is located at PAM+1. (B–D) Engineering the crRNA by introducing an SM at (B) PAM+2, (C) PAM+3, or (D) PAM+4 significantly improves the on-target activity. (E) Fluorescence ratios at different time points demonstrate improved single-nucleotide specificity for BRAF p.V600E detection with limited or no loss of activity. Ratios were calculated by dividing fluorescence signal from reactions spiked with V600E by values measured in reactions spiked with V600 WT. Error bars display the SD of the mean value of triplicate experiments. Activators were added to a final reaction concentration of 50 pM.

Apart from KR105, which is a crRNA designed to detect the mutant p.V600E codon, we designed KR122, a crRNA with full complementarity to the WT *BRAF* p.V600 codon. Since the nucleic acid makeup of liquid biopsies reflects the total somatic genetic material from an individual, largely reflecting WT tissues, KR122 serves as a positive control for *BRAF* CRISPRdx in liquid biopsies and may give an indication of the tumor fraction in a sample. To investigate whether the LbCas12a can discriminate the single-nucleotide difference between these two *BRAF* targets, we assessed the on-target fidelity of the designed crRNAs by spiking WT and mutant activators in separate reactions (Figure 2A). However, using mutant and WT BRAF activators, we observed similar fluorescence curves independent of the supplied activator, indicating that Cas12a was not able to discriminate the single-nucleotide difference, even though the mismatch occurs within the defined seed region.

Previously, crRNA engineering was shown to be a feasible approach toward single-nucleotide specificity in Cas12 CRISPRdx.^22^ One of the strategies includes introducing SMs in proximity to the position of the variant of interest. We introduced mismatches at PAM+2, PAM+3, and PAM+4, all within the Cas12a seed region, yielding crRNAs KR119–KR121 and KR123–KR125 (Figures 2B–2D). None of these introduced mismatches led to a complete loss of crRNA activity, again demonstrating that Cas12a tolerates single mismatches within the seed region. Although the overall activity of the crRNA is slightly decreased, we observed that the SMs significantly reduced off-target activity (Figure 2E). Evidently, having two mismatches within this context is poorly tolerated. Introducing SMs within 5 h of incubation, we found an up to 6-fold and 7.5-fold specificity improvement for V600E and WT detection, respectively. The best result was obtained using KR121 and KR125, which contain an SM at PAM+4. Hence, we selected this crRNA pair to develop a single-nucleotide-specific detection test.

### Engineered crRNAs can determine cell line *BRAF* statuses from culture-derived cell-free DNA

Instead of using rare tissue biopsies, we sought a model to optimize the test further as a minimally invasive liquid biopsy test. As previously demonstrated, the cell-free DNA (cfDNA) obtained from used culture medium shows fragmentation patterns that are highly similar to those of cfDNA found in blood.^11^ We therefore first tested this source of cfDNA to determine whether the engineered crRNAs could properly genotype *BRAF*^*V600*^ statuses.

**Figure 3:**
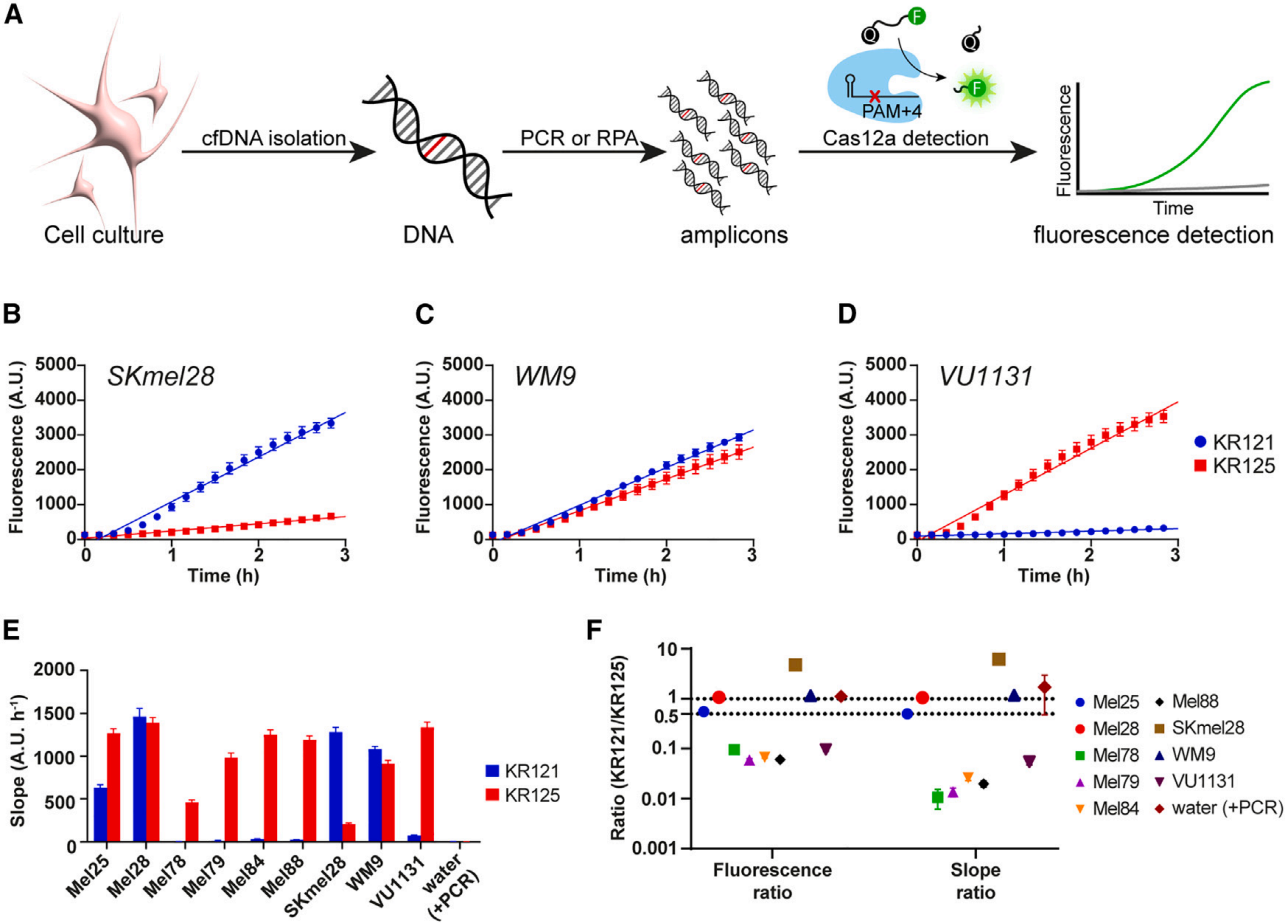
Cas12a distinguishes between heterozygosity and homozygosity for BRAF p.V600E alleles (A) Schematic overview of the derivation of cfDNA from conditioned mammalian cell culture medium. After isolation of shed DNA fragments, nucleic acid amplification enables Cas12-based detection and collateral cleavage of a quenched (Q) fluorescence (F) probe. (B–D) SKmel28 (B), (C) WM9, and (D) VU1131 serve as characterized control cell lines for which the BRAF status and zygosity are known.^24^ Slopes were calculated from the displayed solid regression lines. (E) Regression slopes from CRISPRdx reactions on a melanoma cell line panel with unknown BRAF statuses (Mel25, Mel28, Mel78, Mel79, Mel84, and Mel88). (F) Comparison between fluorescence endpoint ratio at 3 h and regression slope ratio on a logarithmic scale. Regressions were done over 3 h. Ratios were calculated by division normalization for KR125 fluorescence or slope. Reactions display triplicate experiments, and error bars indicate SEMs.

We used dilutions of culture-derived cfDNA that resemble those of actual liquid biopsy cfDNA levels, which are generally low. Moreover, considering the fact that the majority of the cfDNA pool reflects the rest of the genome, the molarity of the *BRAF* activator sequence is expected to be below the amplification-free Cas12a picomolar detection limit.^23^ To overcome this, we introduced a PCR amplification step that enabled attomolar sensitivity (Figure S2D). It is important to realize that cfDNA is highly fragmented—most fragments being ∼166 bp^2^—which can limit the efficiency of PCR, potentially leading to false negatives. Therefore, we used primers to produce a ∼100-bp amplicon. Using this setup (Figure 3A), we tested whether the engineered crRNAs are sufficiently specific to determine the *BRAF* status of characterized cell lines SKmel28, WM9, and VU1131 (Figures 3B–3D).

In line with the known genotypes of these reference cell lines,^24^ SKmel28 and WM9 tested positive and VU1131 tested negative for *BRAF*^*V600E*^. Additionally, using a guide to detect WT *BRAF*, we could correctly identify that SKmel28 is *BRAF*^*V600E*^ homozygous, WM9 is *BRAF*^*V600E*^ heterozygous, and VU1131 is WT *BRAF*^*V600*^ homozygous. This setup worked equally well using recombinase polymerase amplification, an isothermal amplification method that could be useful to make this test more suitable for point-of-care testing (Figure S3).

We next sought to use this setup to identify the *BRAF* status of a panel of cell lines (Mel25, Mel28, Mel78, Mel79, Mel84, and Mel88) with unknown genotypes, derived from metastatic melanoma biopsies (Figure 3E). Mel28 in this panel is not the same as the used *BRAF*^*V600E*^ homozygous reference cell line SKmel28. The assay identified Mel25 and Mel28 as *BRAF*^*V600E*^ mutant cell lines, whereas the rest of the panel did not test positive for this mutation. The outcome for each cell line was confirmed through Sanger sequencing the amplified locus (Figure S4). The *BRAF* WT allele was detected in all cell lines of the panel, suggesting that Mel25 and Mel28 are *BRAF*^*V600E*^ heterozygous and that the rest are WT *BRAF*^*V600*^ homozygous. For both the fluorescence endpoint measurements as well as the calculated slopes, normalizing KR121-signal to KR125-signal gives a ratio score that can be used to classify samples according to their zygosity (Figure 3F). *BRAF*^*V600E*^ homozygous cell lines such as SKmel28 were found with ratio scores of at least 5, and, conversely, homozygous WT cell lines such as VU1131 cluster with ratio scores below 0.1. If the ratio score equals 1, then both KR121 and KR125 contribute equally to the observed fluorescence, and the cell line is heterozygous, which is the case for WM9. Mel78, Mel79, Mel84, and Mel88 had a ratio score below 0.1, suggesting a homozygous WT *BRAF*^*V600*^ status. Mel28 had a ratio score of 1, suggesting *BRAF*^*V600E*^ heterozygosity. All genotypes were confirmed through Sanger sequencing of the amplified locus (Figure S4).

The ratio scores of Mel25 were consistently around 0.5, not around 1, as expected for a heterozygous cell line, suggesting that the cell line is *BRAF*^*V600E+*^, but does not contain an equal amount of WT and mutant alleles (Figure 3F). This could be the case if the cell line harbors more than two copies of the *BRAF* locus, through, for example, gene duplication or aneuploidy (Figure S5A). We performed nanopore sequencing on the amplified locus to determine the fraction of mutant and WT alleles in Mel25, WM9, and VU1131. In line with the known genotypes^24^ of reference cell lines WM9 and VU1131, we found a 1:1 and a 1:0 ratio for WT:mutant alleles, respectively. The NGS data confirmed the heterozygous imbalance in Mel25, for which we found a relatively larger fraction of WT alleles (Figure S5B).

### SMs should be positioned close to the variant of interest

**Figure 4:**
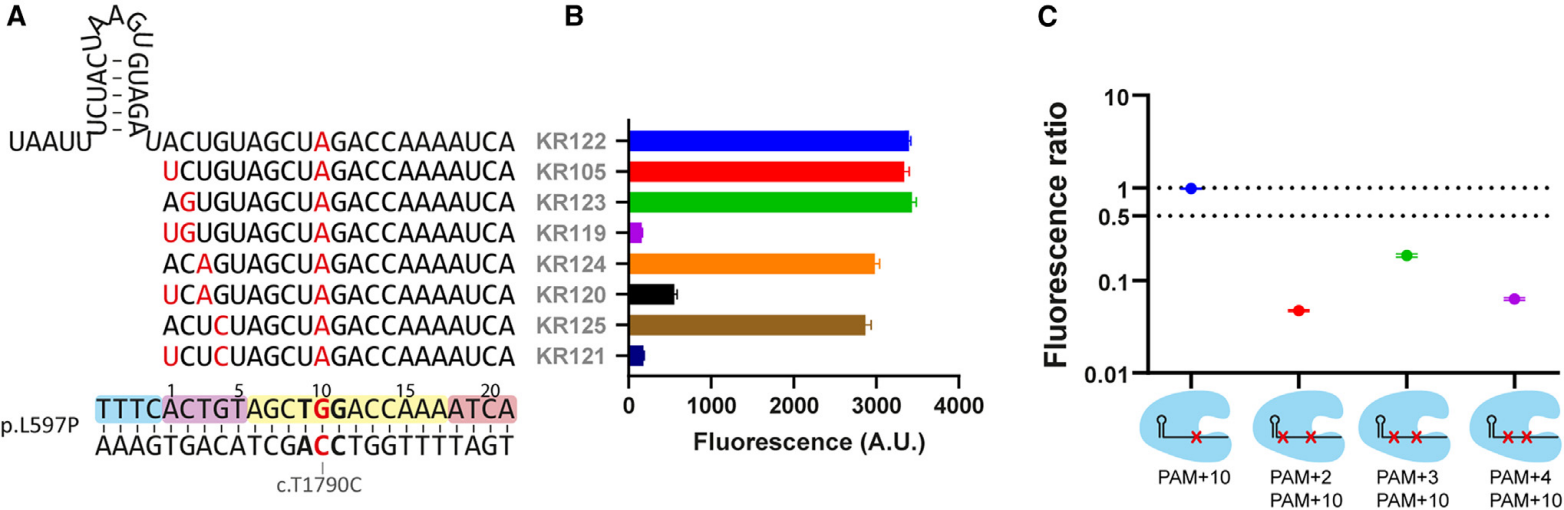
PAM-distal synthetic mismatches do not confer increased specificity for variant recognition within the seed region (A) Overview of 8 different crRNAs with single or dual synthetic mismatches against Mel78 (BRAF^L597P^ homozygous) for the detection of BRAF^V600E^ or BRAF^V600^ at PAM+1. Positions in the target sequence relative to the PAM site have been indicated every five bases. (B) Fluorescence endpoint values after 3 h of incubation. (C) Fluorescence ratio scores calculated from endpoint fluorescence values at 3 h of incubation, plotted on a logarithmic scale. Ratio scores were calculated by normalizing reactions with BRAF^V600E^-detecting crRNAs to reactions with BRAF^V600^-detecting crRNAs. Error bars display the SD of the mean value of triplicate experiments.

We found significantly lower fluorescence values than expected for homozygous WT when determining the *BRAF*^*V600*^ status in Mel78 (Figures 3E andS4). By Sanger analysis, it was confirmed that Mel78 is indeed WT for *BRAF*^*V600*^, yet it contains another variant (c.T1790C) within the target sequence at PAM+10, resulting in pathogenic missense mutation p.L597P (dbSNP: rs121913366). Apparently, this naturally occurring mismatch at a PAM-distal site lowers the detection efficiency, but is still tolerated by KR125 (Figure 4A). If an SM is placed this far outside the seed region, then we expected that it would be too far to increase the specificity, and as a consequence, the original crRNA pair KR105/KR122 would not be able to distinguish between WT and mutant *BRAF*. This was confirmed by repeating the detection on Mel78 (Figure 4B). This suggests that placing the SM should preferably be close to the variant of interest, and ideally within the seed region. Introducing extra SMs within the seed region restored the diagnostic capacity to detect *BRAF*^*V600*^ with fidelity (Figure 4C).

### Cas12a can identify *BRAF*^*V600E*^ in patient plasma samples

**Figure 5:**
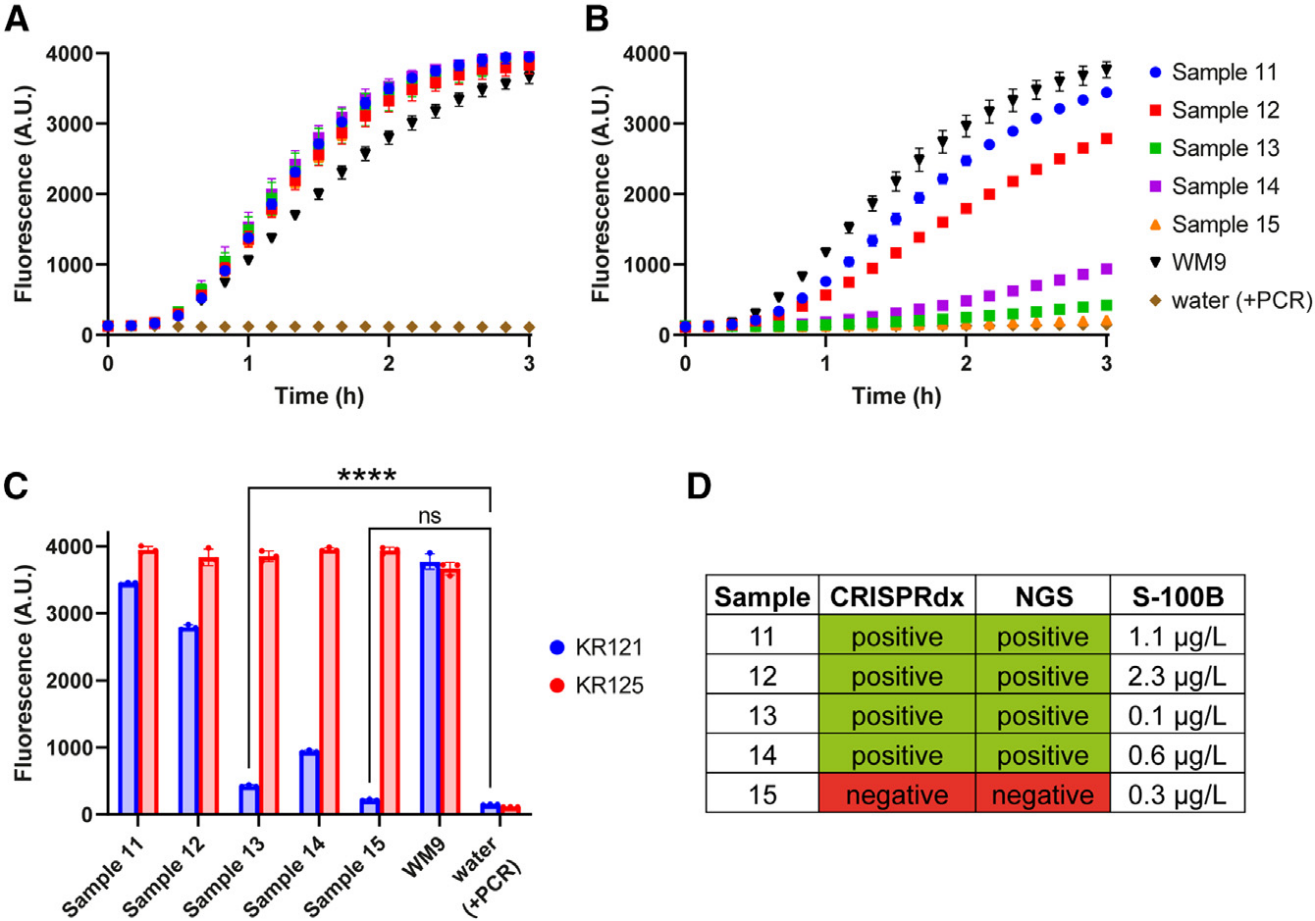
Cas12a identifies BRAF^V600E^-positive sequences in human plasma samples (A and B) Raw fluorescence data of CRISPRdx using (A) KR125 and (B) KR121 over 3 h. Culture-derived cfDNA from WM9, a characterized heterozygous BRAF^V600E^ cell line, was used as control input material. Error bars display SD of the mean of triplicate experiments. (C) Endpoint fluorescence values after 3 h of incubation. All three replicate values are shown, together with error bars displaying the SD. Where relevant, results from a two-way ANOVA comparison between tested samples and the water control have been indicated as ns (non-significant) or as ∗∗∗∗*p* < 0.0001. (D) Overview of the BRAF^V600E^ status of the tested samples as determined by CRISPRdx and NGS, compared with the measured S-100B levels.

As a final proof of principle, we tested five blinded human plasma samples to see whether CRISPRdx could identify which samples contained *BRAF*^*V600E*^. Since the source of cfDNA composition of liquid biopsies includes both tumor and normal tissues, we used KR121 and KR125 to detect both mutant and WT *BRAF*, respectively*.* WT *BRAF* sequences were detected in all plasma samples, with very similar fluorescence growth rates that reached a plateau in about 3 h (Figure 5A). Since the tumor fractions of plasma cfDNA may vary among different cancer stages and patients, we expected a range of fluorescence growth rates when using KR121 to detect *BRAF*^*V600E*^, which we indeed observed (Figure 5B). After 3 h of incubation, we concluded that samples 11, 12, 13, and 14 are *BRAF*^*V600E+*^; of these samples, 11 and 12 showed very strong fluorescence signals, and those of 13 and 14 were much lower (Figure 5C). Comparison with heterozygous control cell line WM9 and a water negative control indicates that sample 15 is *BRAF*^*V600E−*^. For all five plasma samples, the corresponding tissue biopsies were analyzed with NGS to validate our findings, confirming the mutation status found with CRISPRdx for all (Figure 5D). When comparing the S-100B (a common biomarker for melanoma activity) levels, we found very low values for samples 13 and 14, which correspond to the values found using CRISPRdx.

### crRNA seed region engineering refines the detection of other SNVs

**Figure 6:**
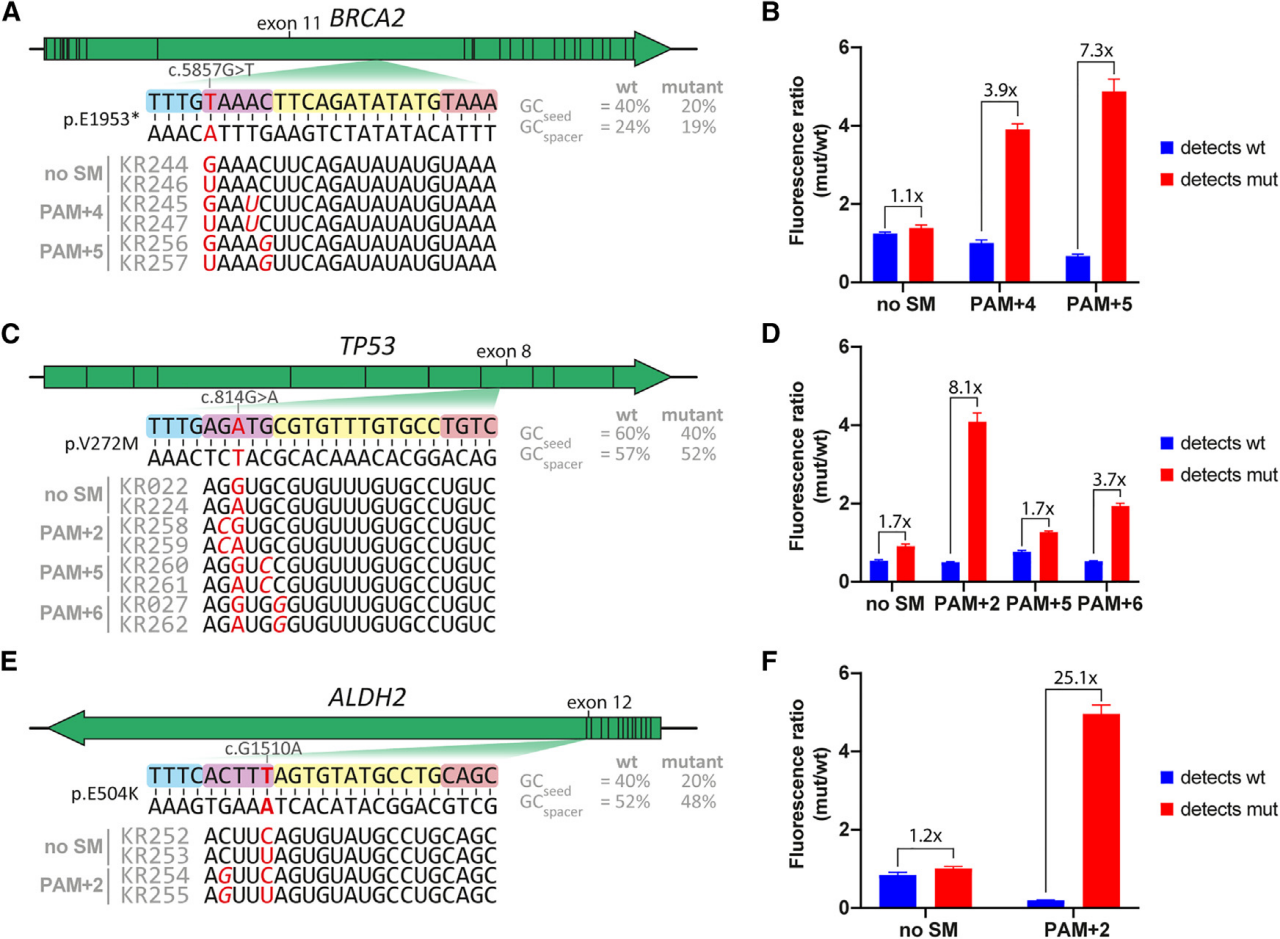
crRNA-based optimization of CRISPRdx on pathogenic seed region mutations in BRCA2, TP53, and ALDH2 (A) Graphic overview of the BRCA2^E1953∗^ target site in exon 11 and a panel of six crRNA spacers. (B) The 3-h endpoint fluorescence ratio per crRNA, calculated from separate CRISPRdx reactions with spiked synthetic mutant or WT BRCA2 dsDNA. (C) Graphic overview of the TP53^V272M^ target site in exon 8 and a panel of eight crRNA spacers. (D) The 3-h endpoint fluorescence ratio per crRNA, calculated from separate CRISPRdx reactions with spiked synthetic mutant or WT TP53 dsDNA. (E) Graphic overview of the ALDH2^E504K^ target site in exon 12 and a panel of four crRNA spacers. (F) The 3-h endpoint fluorescence ratio per crRNA, calculated from separate CRISPRdx reactions with spiked synthetic mutant or WT ALDH2 dsDNA. All target site graphics annotate the GC content of the seed region and of the entire spacer. The 5′ crRNA hairpins are not displayed in the graphic, and synthetic mismatches are indicated with red in italics. Fluorescence ratios were calculated through dividing reactions with mutant DNA (mut) by reactions with WT DNA (wt). Fold changes between fluorescence ratios have been indicated as pairwise comparisons. Error bars indicate the SEM from triplicate experiments. Legends indicate crRNAs and the position of an SM where relevant. Time-dependent fluorescence ratios are shown in Figure S6.

To examine whether crRNA seed engineering also improves specific detection of other relevant targets, we tested three other hits found by ARTEMIS. *BRCA2* p.E1953∗ (c.5857G>T; dbSNP: rs80358814)*, TP53* p.V272M (c.814G>A; dbSNP: rs121912657), and *ALDH2* p.E504K (c.1510G>A; dbSNP: rs671) are cancer-associated SNVs at PAM+1, PAM+3, and PAM+5, respectively, the last of which is claimed to be the most common human point mutation^25^ (Figures 6A, 6C, and 6E). We first investigated whether non-engineered crRNAs are specific enough to distinguish between WT and mutant DNA by calculating the endpoint fluorescence ratios (Figures 6B, 6D, and 6F). crRNAs designed to detect mutant DNA with low off-target activity should result in a ratio ≫ 1, and likewise, crRNAs to detect WT DNA should result in ratios ≪ 1. We found that crRNAs without SMs could not detect any of the variants with sufficient specificity, which is in line with our above-described findings for *BRAF*^*V600E*^ detection at PAM+1 (Figure 2A), and further highlights that single-nucleotide fidelity within the seed region may not be assumed under these circumstances.

Just like for *BRAF*^*V600E*^, introducing a PAM+4 SM improved the *BRCA2*^*E1953∗*^ test specificity. For *BRCA2*^*E1953∗*^, this PAM+4 SM involves disruption of a relatively weak T–A bond, which was also the case for the PAM+3 SM for *BRAF*^*V600E*^ detection, resulting in limited effect and increased background noise (Figure 2C). Disrupting the G–C bond at *BRCA*^*E1953∗*^ PAM+5 improved the distinctive capacity even further. We decided to introduce SMs that disrupt G–C base pairs at *TP53*^*V272M*^ PAM+2 and PAM+5; PAM+5 severely reduced the overall crRNA activity (Figures S6C and S6D). However, the PAM+2 SM strongly improved the specificity of the mutant-detecting crRNA, without loss of signal strength. Finally, although not as effective as the PAM+2 SM, a PAM+6 mismatch did improve the specificity, despite slower kinetics (Figures S6C and S6D). Also for *ALDH2*^*E504K*^, an SM at PAM+2 improved the accuracy of the detection reactions significantly for both the WT and mutant-detecting reactions.

## Discussion

Detection of clinically relevant SNVs is vital for guiding treatment decisions.^18^^,^^19^ Here, we developed an approach for the feasibility of accurate CRISPR-Cas12a-based detection of cancer-associated point mutation p.V600E, the most frequently occurring *BRAF* variant. Our results reveal that single-nucleotide specificity in Cas12a-based diagnostics is not self-evident and requires additional measures. We have previously demonstrated that this can be achieved by tuning the rate of amplification or by choosing alternative Cas12a variants.^11^ In this paper, we demonstrate that introducing an extra mismatch by crRNA seed region engineering is a more efficient solution.

Previously, we demonstrated the feasibility of Cas12a-based detection of *TP53* SNVs.^11^ We speculated that the limited specificity we found could at least partially be explained because the mutations were located downstream of the seed region. Hence, in this study, we focused on the role of specific SNVs within the seed region and investigated whether they could influence the specificity of Cas12a, due to the reported reduced tolerance for mismatches in this region.^7^^,^^26^ Unexpectedly, we found that non-engineered crRNAs did not sense any difference between WT *BRAF* and *BRAF*^*V600E*^, which is a variant located at PAM+1. Similarly, we tested SNVs in *BRCA2*, *TP53*, and *ALDH2* that confirmed the mismatch tolerance of Cas12a in the seed region. These findings are in accordance with those of He and colleagues, which led them to use Cas14a instead.^27^ To our knowledge, the only other instance of Cas12a-based *BRAF*^*V600E*^ CRISPRdx used mutant-specific aptameric capture and consequential release of a different target substrate for RNA-guided detection, circumventing the single-nucleotide specificity issue.^28^ Other instances of achieving single-nucleotide fidelity were achieved using protein engineering,^29^^,^^30^ effector choice,^27^ SNV-specific amplification,^31^ or SNV-specific digestion.^32^ Awareness of potential aspecific Cas12a activity, even within the seed region, is vital to consider when deploying type V effectors for diagnostics or therapeutics. Biologically, the seed region might not discriminate single nucleotides too strictly to allow CRISPR-equipped bacteria to combat invading phages that mutate rapidly, a commonly described “escape route” for phages.^33^

Upon discovery of Cas12a collateral activity in 2018, Chen et al. demonstrated that adjacent double mismatches within the target reduce collateral activity greatly if placed within the PAM-proximal region.^8^ When optimizing a test for a COVID-19 variant, Huang and colleagues introduced the term “synthetic mismatches” for artificially introduced mismatching bases in the Cas12a crRNA.^22^ Their data exemplified how a non-engineered crRNA normally could not detect a PAM+6 point mutation (which is outside the seed region) with single-nucleotide fidelity, but gained specificity by introducing SMs. The biggest gain was found for SMs introduced on the PAM-proximal side of the variant of interest. SM-based strategies were adopted in multiple Cas9,^34^^,^^35^ Cas12,^31^^,^^36^ and Cas13^37^^,^^38^ SNV-detecting CRISPRdx, with varying degrees of improvement. Many of the published assays focus on optimizing high-fidelity detection of an SNV of interest, lacking a generalizable rationale for which position or which base should be mutated. Based on these findings, we engineered our crRNAs by incorporating SMs within the seed region, with the goal of achieving single-nucleotide fidelity detection of pathogenic SNVs, independent of the SNV position or target GC content.

This resulted in a very specific set of crRNAs for all four SNVs tested, without significant loss of signal strength. SMs do not necessarily have to be directly adjacent to the mutation of interest, as long as both are located within the seed region. Two mismatches within the Cas12a seed region are sufficient to largely reduce collateral cleavage activity. In contrast to previous studies, we demonstrate that this approach works independently for all four mutations tested, generalizing this approach as a strategy toward specific genome-wide detection of clinically relevant SNVs. Serendipitously, we explored the impact of a mismatch beyond the seed region on distinguishing an SNV within the seed region (Figure 4). crRNAs designed with ARTEMIS avoid common variants in the PAM site, but do not anticipate unforeseen seed region mutations, which can severely reduce detection efficiency (Figures 2 and 6). These mutations may lead to false negatives and pose an issue for all forms of CRISPR-based testing. Future efforts could explore using a cocktail of crRNAs covering potential mutation sites to reduce the likelihood of missed detections.

Next to the position, our results indicate that the type of mismatch also influences the effect of the SM. Unraveling which mismatch types are least tolerated supports the road to rational crRNA design for high-specificity CRISPRdx. We found that SMs disrupting G–C bonds within the seed often have a greater effect than SMs eliminating T–A bonds. Based on previous findings, we introduced only SMs that result in homopairs (e.g., G–G, C–C) or T∙U. While we have not tested whether these are the most efficient SMs, these combinations avoided potential wobble base pairs, which would contribute to mismatch tolerance.^10^^,^^11^^,^^39^ Our study highlights different aspects to be considered when engineering crRNA SMs. While preparing this manuscript, Molina Vargas and colleagues presented their findings on single-nucleotide fidelity Cas13a CRISPRdx,^40^ which included strategies similar to those we present here for Cas12a. These parallel developments suggest that design strategies for Cas12 (DNA) and Cas13 (RNA) diagnostics may have more in common than originally anticipated, bridging these two diagnostic efforts.

Our results demonstrate that crRNA engineering by introducing SMs is a valid and simple approach to improve CRISPRdx accuracy. However, there are multiple other, often more complex ways to engineer crRNAs—for instance through 3′,2′-*O*-methyl base modifications,^41^ deletions/insertions,^42^ RNA/DNA extensions,^43^ crRNA truncation,^29^ split crRNAs,^44^ RNA-to-DNA substitutions,^45^ base (de)methylation,^46^ and non-canonical/ortholog crRNA structures.^47^ In combination with other factors, such as effector choice, target amplification strategies, and readout choice, crRNA engineering can functionally aid in achieving rapid and high-specificity CRISPRdx.

A second aim of this study was to obtain better insights into what percentage of the human genome is targetable using CRISPR/Cas12a seed regions. Previously, an algorithm was published that identifies targetable PAMs that form due to SNVs.^48^ Whereas this is an elegant way of reducing off-target effects, the amount of targetable clinically relevant SNVs is very small. Here, we present ARTEMIS as an algorithm that identifies clinically relevant SNVs within Cas12a-targetable seed regions. This study focused only on Cas12a seed regions and oncology-relevant point mutations. However, by slightly adapting the algorithm, the same principle could be extrapolated to identify targetable SNVs for other diseases or for different Cas proteins with other PAM preferences, and perhaps in different reference organisms, depending on the application. Moreover, ARTEMIS in combination with SMs may offer a platform to strategize rational crRNA design for CRISPR-based genomic editing (e.g., for gene therapy) with improved accuracy. In this study, ARTEMIS excluded PAMs that overlap with SNPs (allele frequency >1%) to reveal targets suitable for developing a broad-range test. However, this filtering step could be omitted when using a patient’s unique genome as a reference to look for somatic cancer mutations, increasing the range of applications in personalized cancer monitoring. Along the same lines, targeting unique pathogenic fusion gene junctions may expand future personalized CRISPRdx applications. Complementarily, if the molecular kinetics of SNV detection beyond the seed region are better understood, then the underlying strategies can become more widely applicable, expanding the targetability of the pathogenic mutational landscape.

The speed, low costs, and adaptability at which CRISPRdx tests can operate offer great potential for advancing point-of-care (e.g., general practitioner-monitored or home testing) DNA tests, for screening and diagnostics in remote or underdeveloped areas, or as a quick alternative test for verification or monitoring of NGS-based findings. For a reliable test, achieving high accuracy is imperative, particularly when detecting SNVs. We demonstrated that crRNA engineering using SMs significantly enhances Cas12a-based detection accuracy, enabling semi-quantitative allelotyping of cell lines and rapid detection of *BRAF*^*V600E*^ in human plasma samples within 3 h. There are strategies to reduce turnaround time—for instance, optimizing Cas12a reaction conditions such as probe length and buffer composition can increase fluorescence rate up to 50-fold.^49^ Especially in the case of *BRAF*^*V600E*^, such rapid and accurate diagnostics have the potential to support time-efficient decision-making for therapeutics and treatment efficacy monitoring, contributing to improved prognosis and survival. The CRISPRdx signal on patient plasma samples seemed to correspond to the determined S-100B levels. This semi-quantitative correspondence in CRISPRdx signal could be attributed to the amount of detected ctDNA copies reflecting the amount of active melanoma cells, and thereby the S-100B levels. Previously studied correlations between S-100B levels and ctDNA support this hypothesis.^50^^,^^51^^,^^52^ Liquid biopsy testing can benefit from disease-specific insights in cfDNA fragmentomics, targeting loci patterns that are enriched for certain conditions,^53^ or taking ultra-short ctDNA fragments (<100 bp)^54^ into account for improved sensitivity.

Altogether, our data contribute to insights into rational crRNA design to support development toward cost-effective, point-of-care, and high-fidelity cancer diagnostics. Future advancements could leverage SMs in combination with other CRISPRdx strategies such as absolute target quantification,^55^^,^^56^ amplification-free detection,^57^^,^^58^ or point-of-care microfluidics,^59^^,^^60^^,^^61^ depending on the clinical purpose and demand. In combination with tailored crRNA design, these approaches hold promise for early cancer detection and monitoring of treatment efficacy, contributing to affordable and effective personalized healthcare.

### Limitations of the study

ARTEMIS identifies LbCas12a candidate target sites with a 5′ TTTV PAM, and looks for SNVs overlapping with the corresponding pentanucleotide seed region. The inherently stringent PAM preference and the limited range of the seed limit the targetability of genome-wide SNVs. ARTEMIS can easily be adjusted to allow exploring engineered or evolved CRISPR effector variants that will offer broader coverage, enabling detection of SNVs that were filtered out in this study.

Although crRNAs designed with ARTEMIS avoid common variants in the PAM site, they do not anticipate unforeseen seed region mutations, which can severely reduce detection efficiency (Figures 2 and 6), and in the worst cases may lead to false negative outcomes. Future efforts could explore using a cocktail of crRNAs covering potential mutation sites to reduce the likelihood of missed detections.

Next to position, we report that the mismatched base type also impacts the efficiency of SMs. In this study, we only introduced SMs that result in homopairs (e.g., G–G, C–C) or T–U. While we have not tested whether these are the most efficient SMs, these combinations avoided potential wobble base pairs, which would contribute to mismatch tolerance.

## Resource availability

### Lead contact

Further information and resource requests should be directed to the lead contact, Erik Sistermans (e.sistermans@amsterdamumc.nl).

### Materials availability

This study did not generate new unique reagents.

### Data and code availability

• This paper utilized existing publicly available datasets. The SNV data are accessible at the ClinVar database^12^ (https://www.ncbi.nlm.nih.gov/clinvar/). The reference human genome (GRCh38.p14) is accessible at https://www.ncbi.nlm.nih.gov/datasets/genome/GCF_000001405.40/. The information is also listed in the STAR Methods section in the key resources table. An overview of all genome-wide SNV ARTEMIS hits has been deposited at Zenodo at https://doi.org/10.5281/zenodo.14066692 and is publicly available as of the date of publication.
• All original ARTEMIS code has been deposited at Zenodo at https://doi.org/10.5281/zenodo.14066692 and is publicly available as of the date of publication.
• Any additional information required to reanalyze the data reported in this paper is available from the lead contact upon request.

## Acknowledgments

We gratefully acknowledge the invaluable assistance provided by A.E. Katajamäki, whose efforts were instrumental in initiating this study. We thank A.M. Molina Vargas for the fruitful discussions that significantly helped to advance this work. We extend our appreciation to our colleagues from the Cancer Center Amsterdam, Amsterdam UMC Locatie Vrije Universiteit, T.D. de Gruijl, D.A. Stolk, J.G.C. Stolwijk, and D.A.P. Rockx for providing used mammalian cell culture medium. We thank J. de Rooij for feedback and establishing clinical contacts. We also thank the Amsterdam UMC Core Facility Genomics for providing nucleic acid isolation and NGS services, in particular, W.H. Segerink, R.E. Boyer, and D.L.S. Sie for NGS and data processing. No external funding was obtained for this work.

## Author contributions

Conceptualization (equal), software (supporting), methodology (lead), validation (lead), formal analysis (lead), investigation (lead), data curation (lead), writing—original draft (lead), writing—review and editing (equal), visualization (lead) and project administration (lead), K.A.V.K. Software (lead) and data curation (supporting), J.L. Resources (supporting), R.B. Investigation (supporting), L.O.N. Conceptualization (equal), funding acquisition (lead), supervision (equal), and writing—review & editing (equal), E.A.S. and R.M.F.W.

## Declaration of interests

The authors declare no competing interests.

## STAR★Methods

### Key resources table

**Table:** 

REAGENT or RESOURCE	SOURCE	IDENTIFIER
**Biological samples**		

Metastatic melanoma tissue	Baars et al., 2000	N/A

**Chemicals, peptides, and recombinant proteins**		

sodium penicillin	Yamanouchi Pharma	discontinued
streptomycin sulfate	Radiumfarma-Fisiopharma	discontinued
L-glutamine	Invitrogen	Cat#25030081
2-mercaptoethanol	Merck KGaA	Cat#805740
DNase I	Boehringer Mannheim (Roche)	Cat#776-785
Collagenase	Boehringer Mannheim (Roche)	Cat#11088858001
Hanks’ Balanced Salt Solution (HBSS)	Whithaker Bioproducts	Cat#BW10-543F
dimethyl sulfoxide (DMSO)	Merck KGaA	Cat#102952
Roswell Park Memorial Institute (RPMI) medium	Gibco™	Cat#11875093
Iscove’s modified Dulbecco’s medium (IMDM)	Gibco™	Cat#12440053
Dulbecco’s modified Eagle’s medium (DMEM)	Gibco™	Cat#10566016
Fetal Bovine Serum (FBS)	Hyclone	Cat#SH30071.03
DNase	Roche, Basel	Cat#04716728001
SuRE/Cut™ buffer M	Roche	Cat#11417983001
rCutSmart buffer	New England Biolabs	Cat#B6004S
EnGen® LbCas12a	New England Biolabs	Cat#M0653T
dNTP	New England Biolabs	Cat#N0447L

**Critical commercial assays**		

Elecsys® 801	Roche Diagnostics	Cat#8817278190
QIAsymphony®	Qiagen	Cat#9001301; Cat#937236
Qubit™ dsDNA HS kit	Invitrogen	Cat#Q32851
Q5® Hot Start High-Fidelity DNA Polymerase	New England Biolabs	Cat#M0493L
TwistAmp® Liquid Basic RPA kit	TwistDx™	Cat#TALQBAS01
QIAquick PCR Purification Kit	Qiagen	Cat#28104
Mix2Seq	Eurofins	N/A
Nanopore Native Barcoding Kit 24 V14	Oxford Nanopore Technologies plc.	Cat#SQK-NBD114.24
Nanopore flongle flow cell R10.4.1	Oxford Nanopore Technologies plc.	Cat#FLO-FLG114
NEBNext® Companion Module for Oxford Nanopore Technologies® Ligation Sequencing	New England Biolabs	Cat#E7180S

**Deposited data**		

ClinVar database	Landrum et al.,^12^ 2014	N/A
1000 Genomes variant call-set	Zheng-Bradley et al.,^62^ 2017	N/A
Overview of genome wide SNV hits	This paper	Zenodo: https://doi.org/10.5281/zenodo.14066692

**Experimental models: Cell lines**		

Human: cell line SKmel28	ATCC	Cat#HTB-72; RRID: CVCL_0526
Human: cell line WM9	Rockland	Cat#WM9-01-0001; RRID: CVCL_6806
Human cell line VU1131	prof. R. Brakenhoff (Amsterdam UMC)	Cat#VU-SCC-1131; RRID: CVCL_XX18
Human cell line Mel25	prof. T.D. de Gruijl (Amsterdam UMC)	N/A
Human cell line Mel28	prof. T.D. de Gruijl (Amsterdam UMC)	N/A
Human cell line Mel78	prof. T.D. de Gruijl (Amsterdam UMC)	N/A
Human cell line Mel79	prof. T.D. de Gruijl (Amsterdam UMC)	N/A
Human cell line Mel84	prof. T.D. de Gruijl (Amsterdam UMC)	N/A
Human cell line Mel88	prof. T.D. de Gruijl (Amsterdam UMC)	N/A

**Oligonucleotides**		

**Oligonucleotides**	This paper	See Table S1

**Software and algorithms**		

BCFtools	Danecek et al., 2021	N/A
BEDTools	Quinlan et al., 2010	N/A
i-control™	Tecan Group Ltd.	v2.0
Prism	GraphPad Software LLC.	v10.2.0
ARTEMIS code	This paper	Zenodo: https://doi.org/10.5281/zenodo.14066692

**Other**		

Infinite® plate reader	Tecan Group Ltd.	Infinite 200 Pro M Plex

### Experimental model and study participant details

#### Cell culture

Human melanoma cell lines SKmel28 (*BRAF*^*V600E/V600E*^) and WM9 (*BRAF*^*V600E/wt*^) were cultured in Iscove’s modified Dulbecco’s medium (IMDM), complemented with 10% fetal bovine serum (FBS). VU1131, a *BRAF*^*wt/wt*^ floor of mouth squamous cell carcinoma cell line, was cultured in Dulbecco’s modified Eagle’s medium (DMEM), complemented with 8% FBS. SKmel28, WM9 and VU1131 were used as control cell lines, with *BRAF*^*V600E/V600E*^, *BRAF*^*V600E/wt*^ and *BRAF*^*wt/wt*^ genotypes respectively.^24^ The panel of melanoma cell lines Mel25, Mel28, Mel78, Mel79, Mel84 and Mel88 with unknown *BRAF* statuses was cultured in Roswell Park Memorial Institute 1640 medium (RPMI-1640), complemented with 10% FBS. All media used for culturing melanoma cell lines were complemented with 100 IU/mL sodium penicillin (Yamanouchi Pharma), 100 μg/mL streptomycin sulfate (Radiumfarma-Fisiopharma, Italy), 2 mM L-glutamine (Invitrogen, Thermo Fisher Scientific, Waltham, USA), 50 μM 2-mercaptoethanol (Merck KGaA, Darmstadt Germany). Cells were cultured at 37°C and 5% CO_2_ in a stationary Heracell 240i incubator (Thermo Fisher Scientific) until confluent. Medium was aspirated and spun down at 500 g, followed by storing the supernatant at −20°C until further use for nucleic acid extraction.

#### Patient material & consent

Melanoma cell lines Mel25, Mel28, Mel78, Mel79, Mel84 and Mel88 were generated from metastatic tumor samples collected from advanced-stage patients in accordance with the Helsinki Declaration of 1975. These patients were enrolled under informed written consent in an institutional review board (IRB)-approved clinical study of autologous whole-cell vaccination at the VU University medical center between 1987 and 1998.^63^ Cell suspensions were processed and cryopreserved within 24h of surgical removal as described.^64^ The collected tumor tissue was minced with a scalpel and dissociated 3 times for 45 min with 0.02% DNase I (Boehringer Mannheim, Germany) and 0.14% collagenase (Boehringer Mannheim) in Hanks’ Balanced Salt Solution (HBSS, Whittaker Bioproducts, Walkerville, USA). The cells were cryopreserved in HBSS with 10% dimethyl sulfoxide (DMSO; Merck KGaA) using a controlled rate freezing system and stored in liquid nitrogen. Cryopreserved cell suspensions were thawed in RPMI +10% FBS medium (Hyclone, Thermo Fisher Scientific), supplemented with 5 μg/mL DNase (Roche, Basel, Switzerland), washed and cultured in medium as described above. Melanoma identity was confirmed by flow cytometry-based staining for Melanoma-associated Chondroitin Sulfate Proteoglycan (MCSP).

Patient samples were obtained from K2EDTA-plasma, left over from routine patient care after centrifugation at 2500xg for 5 min at room temperature and stored at −20°C until use. For proof-of-concept, anonymized samples from two *BRAF*^V600E^-positive patients and one *BRAF*^V600E^-negative patient were used. S-100B levels are routinely monitored as an indicator of disease activity in these patients with melanoma, which was determined using an electrochemiluminescence immunoassay (Elecsys 801, Roche Diagnostics). Paired NGS data corresponds to biopsy testing at the time of diagnosis, preceding the routine patient care.

### Method details

#### Nucleic acid preparation & amplification

All synthetic DNA and RNA oligonucleotides were ordered from Integrated DNA Technologies, Inc. (IDT, Coralville, USA) and their sequences are listed in Table S1. dsDNA activators were formed by mixing complementary synthetic ssDNA oligonucleotides in equimolar amounts in 1x SuRE/Cut buffer M (Roche). The mixture was heated to 95°C for 2 min and cooled down 0.1 °C/s to ambient temperature in order to form duplex DNA. The solution was further diluted to desired levels and stored at −20°C until further use. Ordered crRNAs were reconstituted in nuclease-free water to 100μM and stored at −20°C until further use.

Nucleic acids were extracted from cell culture medium or from patient liquid biopsies as described previously.^11^ In brief, we used QIAsymphony (Qiagen, Venlo, The Netherlands) for automated nucleic acid extraction from thawed cell culture medium supernatant or liquid biopsy plasma samples and subsequently quantified the pooled isolates using the Qubit dsDNA HS kit (Invitrogen, Thermo Fisher Scientific) on a Qubit 4 Fluorometer (Invitrogen, Thermo Fisher Scientific). Isolates were kept at −20°C until further use.

*BRAF* exon 15 was PCR amplified with primers KD217, KD219 and Q5 Hot Start High-Fidelity DNA Polymerase (New England Biolabs, Ipswich, USA) according to the manufacturer’s instructions. Alternatively, amplification was done isothermally, using TwistAmp Liquid Basic RPA kit (TwistDx Ltd, UK) with primers KD117 and KD118, for 20 min at 37°C, following 2 min heat inactivation at 80°C. Both amplification methods were done using deoxyribonucleotide triphosphate (dNTP) solution mix (New England Biolabs).

#### Sequencing

For Sanger sequencing, amplicons were purified from PCR or RPA reactions using the QIAquick PCR Purification Kit (Qiagen), eluting in 35 μL nuclease-free water. Sequencing reactions using the Mix2Seq kit (Eurofins Scientific SE, Luxembourg) were prepared according to supplier’s instructions, complemented with one of the primers used for prior amplification. Nanopore NGS library preparation was done with Nanopore V14 chemistry and the NEBNext Companion Module for Oxford Nanopore Technologies Ligation Sequencing (New England Biolabs). Sequencing was done with Nanopore Flongle version R10.4.1 (Oxford Nanopore Technologies plc, UK). Purified PCR samples were barcoded with the Nanopore native Barcoding kit 24 kit (Oxford Nanopore Technologies plc) and sequenced with at least 200.000x coverage. Nanopore sequencing base-calling was done with Dorado v0.5.0 SUP Model 4.3 (https://github.com/nanoporetech/dorado/releases/tag/v0.5.0), with alignment options activated for GRCh38. Data analysis was done using BCFtools^65^ to obtain the polymorphism ratios at position Chr7:140753336-140753336 (*BRAF* exon 15, c.1799).

#### Fluorescent reporter *trans*-cleavage assays

*Trans-*cleavage reactions were carried out in 20 μL final volume in a Low Volume 384-well Black Flat Bottom Polystyrene NBS Microplate (Corning, Corning, USA), as described previously.^11^ Reactions contained 1 × rCutSmart buffer (New England Biolabs), 100 nM ssDNA probe labeled with an Iowa Black-quenched 6-carboxyfluorescein (6-FAM) dye, 40nM EnGen LbCas12a (New England Biolabs) and 50nM crRNA, unless otherwise denoted. Reactions were initiated by adding short synthetic dsDNA activators, or by adding 2 μL of a raw PCR or RPA reaction mixture. Combinatorial assays were done as previously described.^11^ Omitted components were replaced by water to reach 20 μL final volume, keeping the concentrations of the other components constant. Reactions were incubated for an indicated amount of time at 37°C while monitoring fluorescence (λ_ex_ = 485 nm, λ_em_ = 535 nm) every 5 min in an Infinite 200 Pro M Plex plate reader (Tecan Group Ltd., Männedorf, Switzerland). Specific details on plate reader settings are listed in Table S2.

#### ARTEMIS bioinformatics

Pathogenic SNVs were extracted from the Clinvar database^12^ (Clinical significance is ‘Pathogenic’; build 2021-07-31) using BCFtools.^65^ Selection of cancer-associated pathogenic variants was performed by a case-insensitive scan for the occurrence of the following words in the info field: “Cancer”, “Carcinoma”, “Leukemia”, “Leukemia”, “Lymphoma”, “Blastoma”, “Sarcoma”, “Cytoma”, “Glioma”, “Adenoma”, “Tumor”, “Melanoma”, “Thelioma”, “Fibroma”, “Neoplasm”, “Myoma” and “Canthoma”. Python (v3.6.8) was used to scan the human genome (build GRCh38.p14) for the Cas12a TTTV PAM-site motifs using the regular expression (TTT[A,C,G])|([C,G,T]AAA). Seed sites were defined as the 5bp downstream of the detected PAM sites, taking into account the strand orientation of the respective PAM site. Bedtools^66^ (v2.29.2) was used to exclude PAM sites that contain common variants to exclude influences thereof on PAM site recognition. Common variants were defined as variants with an allele frequency of more than 1% in the thousand genomes variant call-set.^62^ Seed regions were merged with Bedtools to prevent multiple sites from overlapping when calculating what part of the human genome is Cas12a-targetable within the seed region. A variant call format file^67^ with pathogenic variants that intersected with these sites was subsequently annotated with the location of the nearest PAM site and CRISPR-target sequencing using pysam (https://github.com/pysam-developers/pysam). Spreadsheet S1 contains an overview of all cancer-associated SNV hits. An overview of all genome-wide SNV hits is available upon request.

### Quantification and statistical analysis

The experiments described in this study were performed as three replicates (*n* = 3), and the figures display mean values ±SD, unless otherwise denoted. Where relevant, significance testing was performed as a two-way ANOVA comparison between tested samples (∗∗∗∗ = *p* < 0.0001). Details on normalization are indicated in the figures or figure legends.

## Supplemental information

Document S1. Figures S1–S6 and Tables S1 and S2

Data S1. Overview of all cancer-associated SNV hits found by ARTEMIS, related to Figure 1

Document S2. Article plus supplemental information
