# Supplementary material for: Synthetic mismatches enable specific CRISPR-Cas12a-based detection of genome-wide SNVs tracked by ARTEMIS
Source: Cell Rep Methods. 2024 Dec 6;4(12):100912. doi: 10.1016/j.crmeth.2024.100912 (PMC11704620; doi:10.1016/j.crmeth.2024.100912)
Supplement: Document S1. Figures S1–S6 and Tables S1 and S2 [file mmc1.pdf]

Cell Reports Methods, Volume *4*

Supplemental information

Synthetic mismatches enable specific CRISPR-Cas12a-based detection of genome-wide SNVs tracked by ARTEMIS

Kavish A.V. Kohabir, Jasper Linthorst, Lars O. Nooi, Rick Brouwer, Rob M.F. Wolthuis, and Erik A. Sistermans

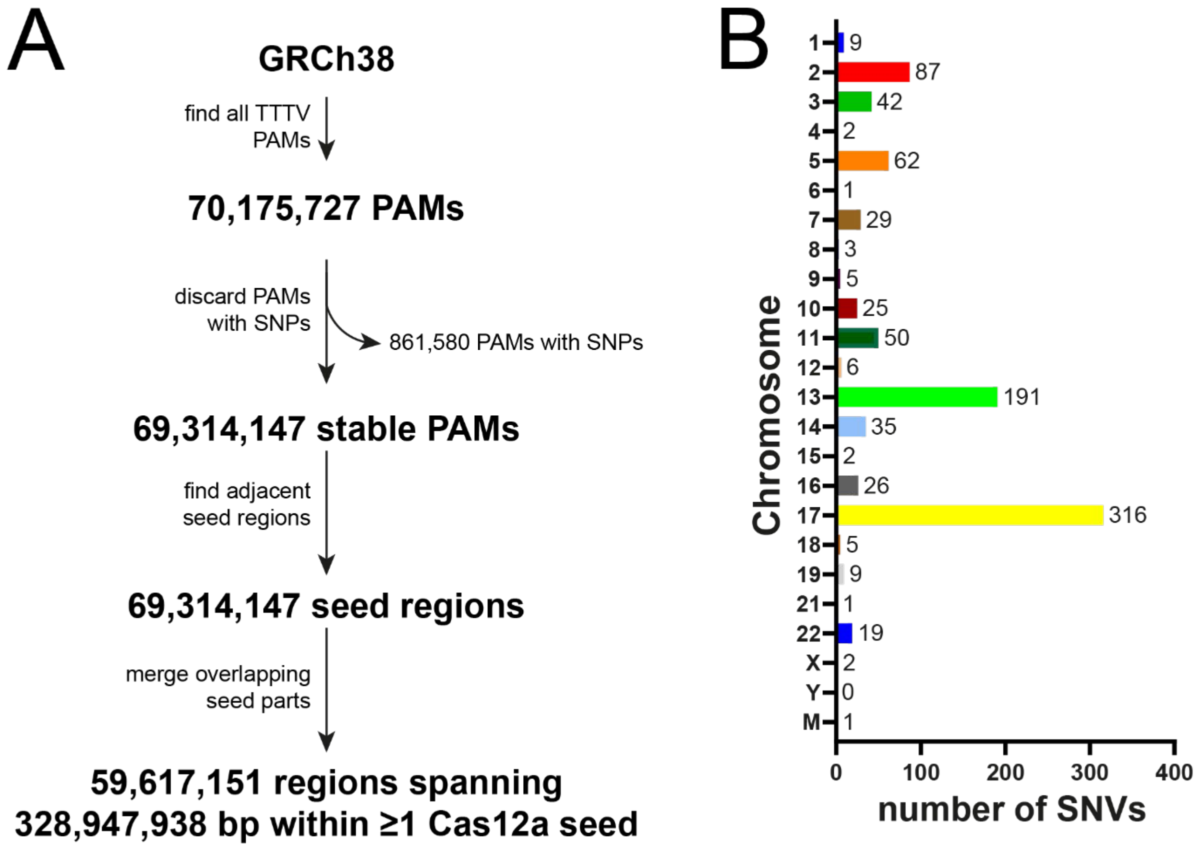

Supplementary Figure S1. Graphic overview of ARTEMIS output for cancer-associated SNVs. Related to Figure 1. (A) Overview of ARTEMIS pipeline and the results for finding unique targetable sites in the human genome within Cas12a seed regions. (B) Number of cancer-associated Cas12a seed-overlapping pathogenic SNVs per chromosome.

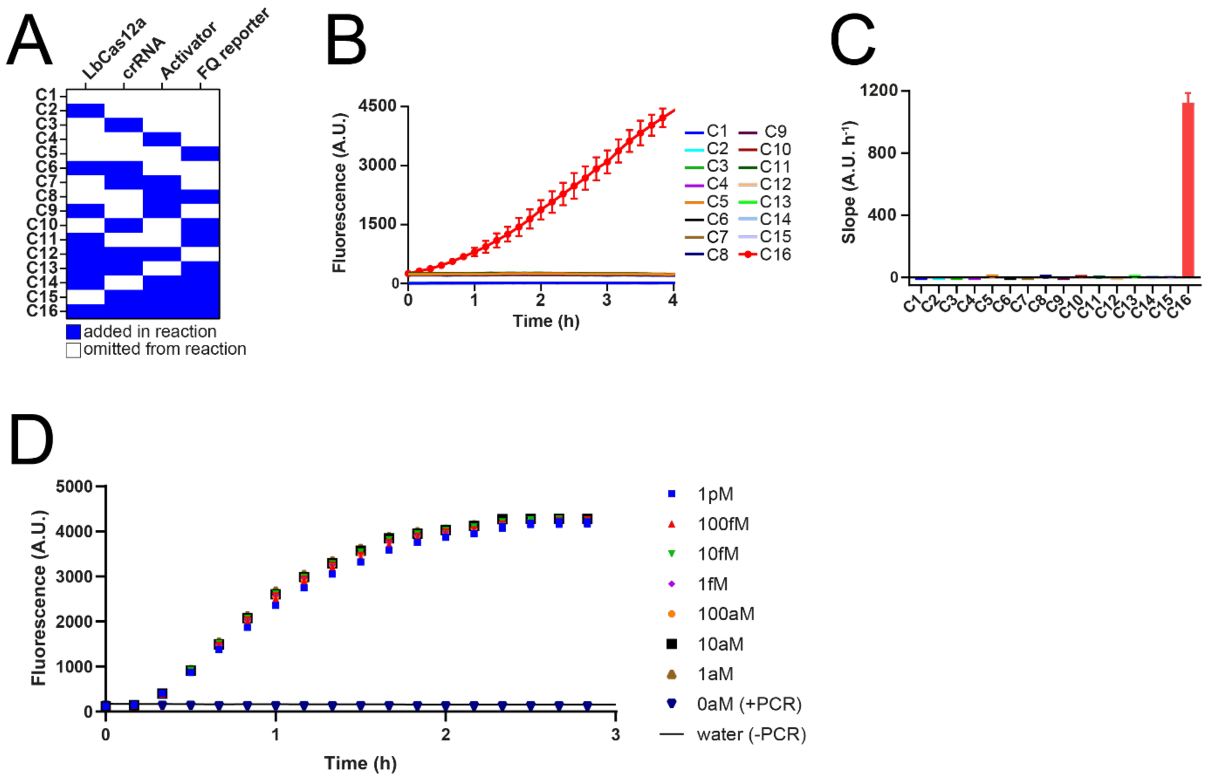

Supplementary Figure S2. Calibrating Cas12a-based detection of *BRAF* p.V600E. Related to Figure 2. (A) Overview of all possible combinations of the four reaction components, with only reaction C16 containing all four components required for activator-dependent collateral cleavage of a quenched fluorescent probe. (B) Fluorescence signal increase over time. Only reaction C16 produces significant fluorescence increase over time. All reactions containing the fluorescent probe (i.e. C5, C8, C10, C11, C13, C14, C15, C16) have a slight offset above background control reaction C1 containing none of the components. (C) Curve slopes as calculated from [B] demonstrate significant fluorescence increase in reaction C16, compared to all other tested reaction compositions (C1-C15). (D) Sensitivity assay with serial dilution of synthetic dsDNA $BRAF^{V600E}$ target serial dilution after PCR amplification demonstrated attomolar sensitivity compared to background reactions. All graphs represent mean values of triplicate experiments and error bars indicate standard deviation of the mean. KR105 was used in reactions containing a crRNA, and annealed 90bp synthetic dsDNA with V600E mutation was used as activator in the designated reactions.

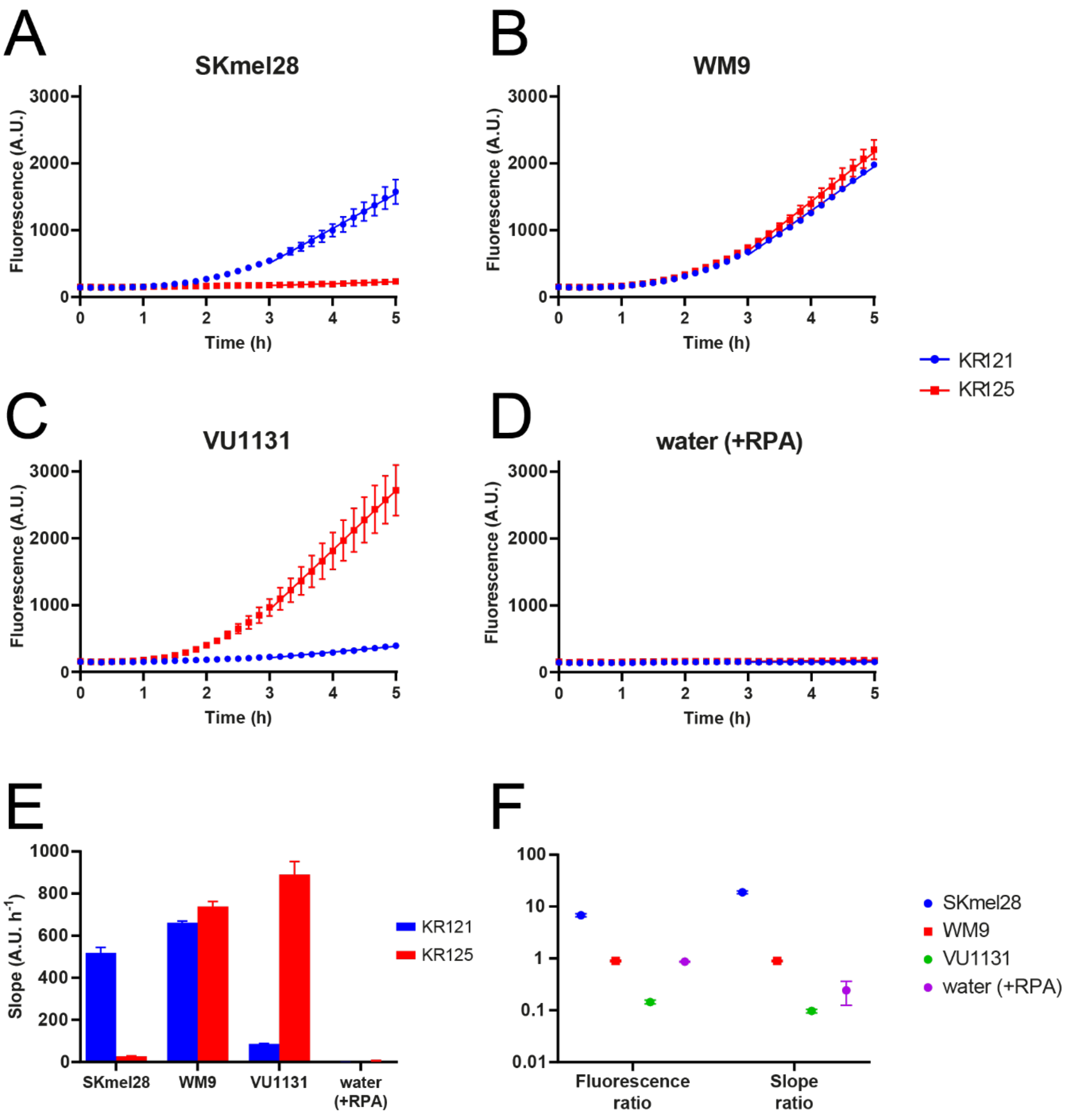

Supplementary Figure S3. Detecting *BRAF* p.V600E in culture-derived cfDNA from reference cell lines after 45min isothermal RPA. Related to Figure 3. Culture-derived cfDNA from (A) SKmel28, (B) WM9 and (C) VU1131, as well as a (D) negative control, water, was used for RPA pre-amplification at 37 degrees prior to CRISPRdx with KR121 & KR125. Graphs represent triplicate fluorescence experimental data. (E) Slopes calculated from linear regression lines in (A-D) between t = 3h and t = 5h. (F) Comparison of fluorescence ratios and slope ratios for each tested cell line on a logarithmic scale. Ratios were calculated by normalized KR121 signal for KR125 signal. Error bars display standard deviation of the mean.

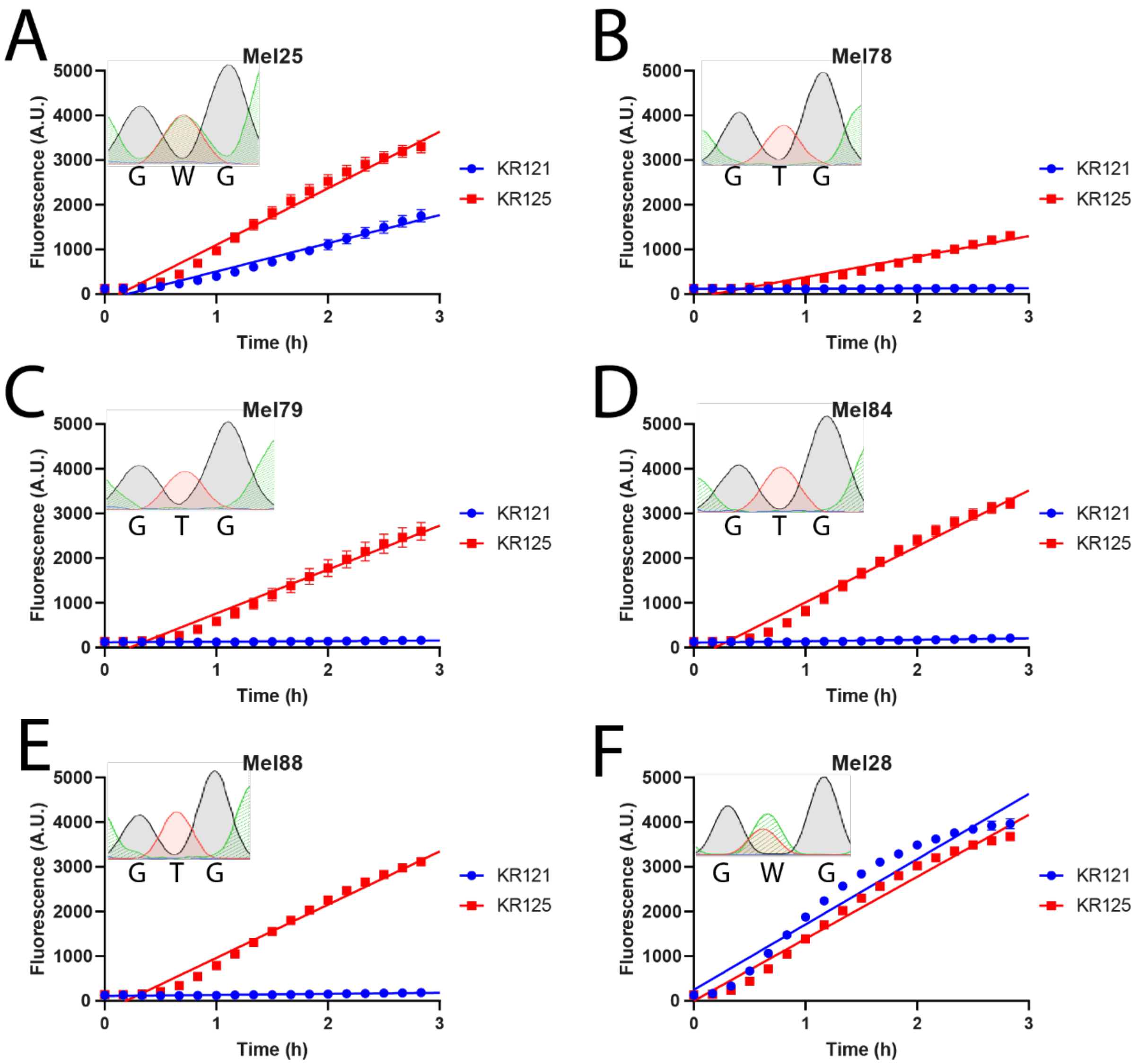

Supplementary Figure S4. Raw CRISPRdx fluorescence data and corresponding Sanger peaks for *BRAF* codon 600 in 6 uncharacterized cell lines. Related to Figure 3. Culture-derived cfDNA derived from (A) Mel25, (B) Mel78, (C) Mel79, (D) Mel84, (E) Mel88 and (F) Mel28 was used for CRISPRdx over a time window of 3 hours. Error bars indicate standard error of the mean. Solid lines indicate results from the regression analysis.

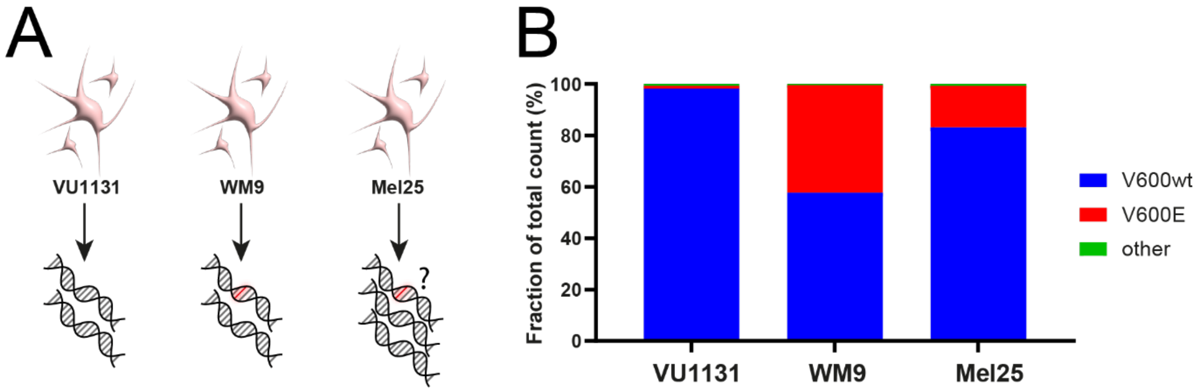

Supplementary Figure S5. NGS analysis of VU1131, WM9 and Mel25. Related to Figure 3. (A) graphic overview of homozygous wild-type VU1131 and heterozygous WM9. Mel25 is expected to have less mutant alleles than wild-type alleles. (B) Normalized allelotypes, sequenced from amplified cfDNA derived from cell culture medium. Normalization was done to the total amount of reads per cell line. Data represent NGS nanopore sequencing runs with at least 200.000x coverage.

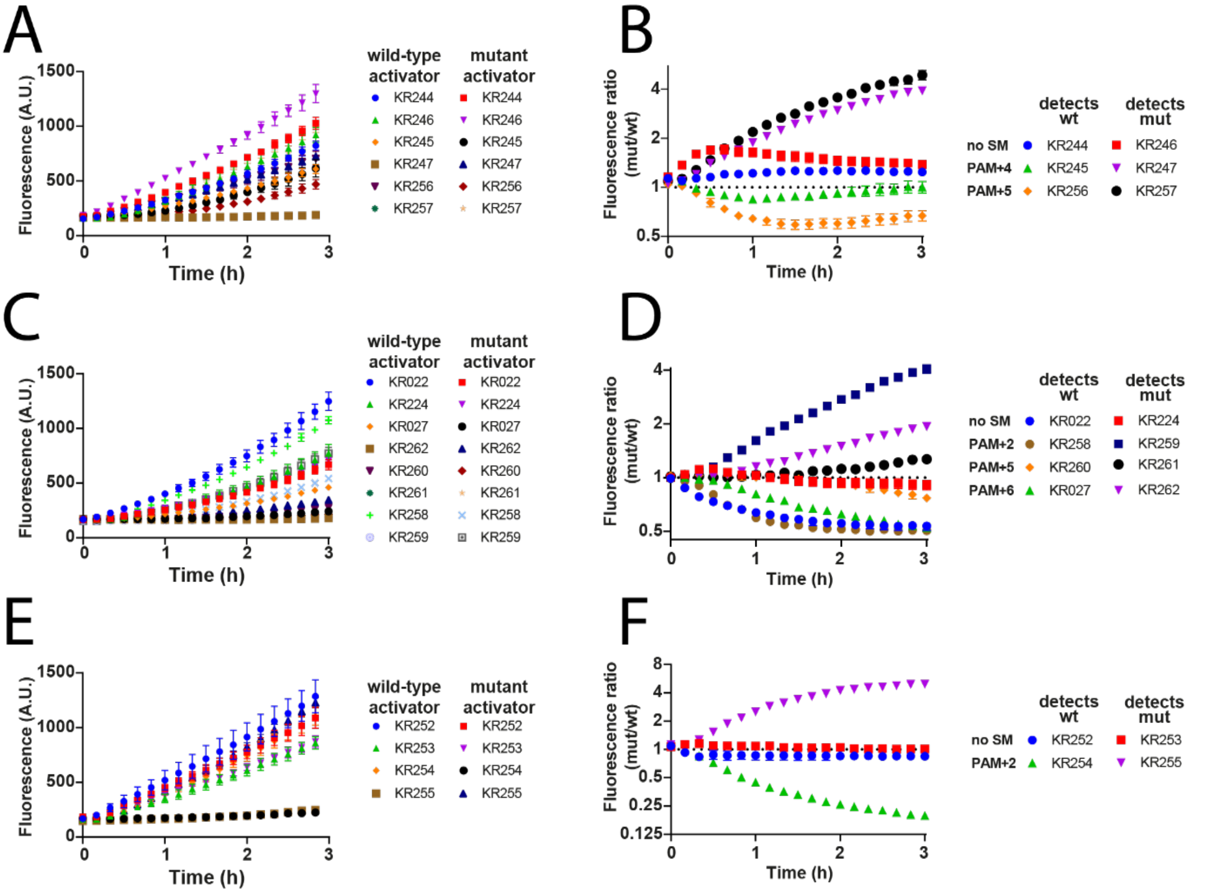

Supplementary Figure S6. Time-dependent fluorescent ratios of CRISPRdx on pathogenic seed region mutations in *BRCA2, TP53* and *ALDH2*. Related to Figure 6. (A) Raw CRISPRdx fluorescence data for reactions spiked with synthetic wild-type or mutant *BRCA2* dsDNA. (B) Time-dependent fluorescence ratio per crRNA, calculated from separate CRISPRdx reaction with spiked synthetic mutant or wild-type *BRCA2* dsDNA. (C) Raw CRISPRdx fluorescence data for reactions spiked with synthetic wild-type or mutant *TP53* dsDNA. (D) Time-dependent fluorescence ratio per crRNA, calculated from separate CRISPRdx reaction with spiked synthetic mutant or wild-type *TP53* dsDNA. (E) Raw CRISPRdx fluorescence data for reactions spiked with synthetic wild-type or mutant *ALDH2* dsDNA. (F) Time-dependent fluorescence ratio per crRNA, calculated from separate CRISPRdx reaction with spiked synthetic mutant or wild-type *ALDH2* dsDNA. Fluorescence ratios were calculated through dividing reactions with mutant DNA (mut) by reactions with wild-type DNA (wt). Error bars indicate standard error of the mean from triplicate experiments. Legends indicate crRNAs and the position of a synthetic mismatch (SM) where relevant.

Supplementary Table S1. Oligonucleotides used in this study. Related to STAR Methods – Method details: Nucleic acid preparation & amplification. All oligonucleotides were ordered from Integrated DNA Technologies (IDT). Coding of chemical modifications and/or proprietary groups are as provided by the manufacturer. Annealing parts of crRNAs and relevant mutations are underlined and indicated in red respectively.

Oligonucleotides used for amplification			
**Oligo no.**	**Name**	**Sequence (5'-->3')**	**length**
KD117	BRAF RPA fw	TGAAGACCTCACAGTAAAAATAGGTGATTT	30 nt
KD118	BRAF RPA rv	CCAGACAACTGTTCAAACTGATGGGACCCA	30 nt
KD128	BRAFV600 Sanger fw	CCAAGTCAATCATCCACAGA	20 nt
KD129	BRAFV600 Sanger rv	ATGGATCCAGACAACTGTTC	20 nt
KD217	BRAF exon15 fw	TTTCTTCATGAAGACCTCAC	20 nt
**Complementary oligonucleotides used for creating activator sequences**			
**Oligo no.**	**Name**	**Sequence (5'-->3')**	**length**
KD113	BRAF V600wt fw	TGAAGACCTCACAGTAAAAATAGGTGATTTTGGTCTAGCTACAGT GAAATCTCGATGGAGTGGGTCCCATCAGTTTGAACAGTTGTCTGG	90 nt
KD114	BRAF V600wt rv	CCAGACAACTGTTCAAACTGATGGGACCCACTCCATCGAGATTTC ACTGTAGCTAGACCAAAATCACCTATTTTTACTGTGAGGTCTTCA	90 nt
KD115	BRAF V600E fw	TGAAGACCTCACAGTAAAAATAGGTGATTTTGGTCTAGCTACAGA GAAATCTCGATGGAGTGGGTCCCATCAGTTTGAACAGTTGTCTGG	90 nt
KD116	BRAF V600E rv	CCAGACAACTGTTCAAACTGATGGGACCCACTCCATCGAGATTTC TCTGTAGCTAGACCAAAATCACCTATTTTTACTGTGAGGTCTTCA	90 nt
KD240	BRCA2 E1953wt fw	accttgtgatgttagtttggaaacttcagatatatgtaaatgtag tatagggaag	55 nt
KD241	BRCA2 E1953wt rv	cttccctatactacatttacatatatctgaagtttccaaactaac atcacaaggt	55 nt
KD242	BRCA2 E1953* fw	accttgtgatgttagtttgTaaacttcagatatatgtaaatgtag tatagggaag	55 nt
KD243	BRCA2 E1953* rv	cttccctatactacatttacatatatctgaagtttAcaaactaac atcacaaggt	55 nt
KD001	TP53_R273wt _fw	AATTCACGGAACAGCTTTGAGGTG**CGT**GTTTGTGCCTGTCCTGGG GGTAC	50 nt
KD002	TP53_R273wt _rv	CCCCAGGACAGGCACAAACACGCACCTCAAAGCTGTTCCGTG	42 nt
KD222	TP53_V272M_ fw	AATTCACGGAACAGCTTTGAGATGCGTGTTTGTGCCTGTCCTGGG GGTAC	50 nt
KD223	TP53_V272M_ rv	CCCCAGGACAGGCACAAACACGCATCTCAAAGCTGTTCCGTG	42 nt
KD248	ALDH2 E504wt fw	gttgggcgagtacgggctgcaggcatacactgaagtgaaaactgt gagtgtggga	55 nt
KD249	ALDH2 E504wt rv	tcccacactcacagttttcacttcagtgtatgcctgcagcccgta ctcgcccaac	55 nt
KD250	ALDH2 E504K fw	gttgggcgagtacgggctgcaggcatacactAaagtgaaaactgt gagtgtggga	55 nt
KD251	ALDH2 E504K rv	tcccacactcacagttttcacttTagtgtatgcctgcagcccgta ctcgcccaac	55 nt

LbCas12a crRNAs

Oligo no.	Name	Sequence (5'-->3')	length
KR105	BRAF V600E	/AltR1/UAAUUUCUACUAAGUGUAGAUU CUGUAGCUAGACCAAAAUCA/AltR2/	42 nt
KR119	BRAF V600E (PAM+2)	/AltR1/UAAUUUCUACUAAGUGUAGAUU GUGUAGCUAGACCAAAAUCA/AltR2/	42 nt
KR120	BRAF V600E (PAM+3)	/AltR1/UAAUUUCUACUAAGUGUAGAUU CAGUAGCUAGACCAAAAUCA/AltR2/	42 nt
KR121	BRAF V600E (PAM+4)	/AltR1/UAAUUUCUACUAAGUGUAGAUU CUCUAGCUAGACCAAAAUCA/AltR2/	42 nt
KR122	BRAF V600wt	/AltR1/UAAUUUCUACUAAGUGUAGAUA CUGUAGCUAGACCAAAAUCA/AltR2/	42 nt
KR123	BRAF V600wt (PAM+2)	/AltR1/UAAUUUCUACUAAGUGUAGAUA GUGUAGCUAGACCAAAAUCA/AltR2/	42 nt
KR124	BRAF V600wt (PAM+3)	/AltR1/UAAUUUCUACUAAGUGUAGAUA CAGUAGCUAGACCAAAAUCA/AltR2/	42 nt
KR125	BRAF V600wt (PAM+4)	/AltR1/UAAUUUCUACUAAGUGUAGAUA CUCUAGCUAGACCAAAAUCA/AltR2/	42 nt
KR027	P53 R273G	/AltR1/UAAUUUCUACUAAGUGUAGAUA GGUGGGUGUUUGUGCCUGUC/AltR2/	42 nt
KR224	P53 V272M	/AltR1/UAAUUUCUACUAAGUGUAGAUA GAUGCGUGUUUGUGCCUGUC/AltR2/	42nt
KR244	BRCA2 E1953wt	/AltR1/UAAUUUCUACUAAGUGUAGAUG AAACUUCAGAUAUAUGUAAA/AltR2/	42 nt
KR245	BRCA2 E1953wt (PAM+4)	/AltR1/UAAUUUCUACUAAGUGUAGAUG AAUCUUCAGAUAUAUGUAAA/AltR2/	42 nt
KR246	BRCA2 E1953*	/AltR1/UAAUUUCUACUAAGUGUAGAUU AAACUUCAGAUAUAUGUAAA/AltR2/	42 nt
KR247	BRCA2 E1953* (PAM+4)	/AltR1/UAAUUUCUACUAAGUGUAGAUU AAUCUUCAGAUAUAUGUAAA/AltR2/	42 nt
KR252	ALDH2 E504wt	/AltR1/UAAUUUCUACUAAGUGUAGAUA CUUCAGUGUAUGCCUGCAGC/AltR2/	42 nt
KR253	ALDH2 E504K	/AltR1/UAAUUUCUACUAAGUGUAGAUA CUUUAGUGUAUGCCUGCAGC/AltR2/	42 nt
KR254	ALDH2 E504wt (PAM+2)	/AltR1/UAAUUUCUACUAAGUGUAGAUA GUUCAGUGUAUGCCUGCAGC/AltR2/	42 nt
KR255	ALDH2 E504K (PAM+2)	/AltR1/UAAUUUCUACUAAGUGUAGAUA GUUUAGUGUAUGCCUGCAGC/AltR2/	42 nt
KR256	BRCA2 E1953wt (PAM+5)	/AltR1/UAAUUUCUACUAAGUGUAGAUG AAAGUUCAGAUAUAUGUAAA/AltR2/	42 nt
KR257	BRCA2 E1953E (PAM+5)	/AltR1/UAAUUUCUACUAAGUGUAGAUU AAAGUUCAGAUAUAUGUAA A/AltR2/	42 nt
KR258	TP53 V272wt (PAM+2)	/AltR1/UAAUUUCUACUAAGUGUAGAUA CGUGCGUGUUUGUGCCUGUC/AltR2/	42 nt
KR259	TP53 V727M (PAM+2)	/AltR1/UAAUUUCUACUAAGUGUAGAUA CAUGCGUGUUUGUGCCUGUC/AltR2/	42 nt
KR260	TP53 V272wt (PAM+5)	/AltR1/UAAUUUCUACUAAGUGUAGAUA GGUCCGUGUUUGUGCCUGUC/AltR2/	42 nt
KR261	TP53 V727M (PAM+5)	/AltR1/UAAUUUCUACUAAGUGUAGAUA GAUCCGUGUUUGUGCCUGUC/AltR2/	42 nt
KR262	TP53 V272M (PAM+6)	/AltR1/UAAUUUCUACUAAGUGUAGAUA GAUGGGUGUUUGUGCCUGUC/AltR2/	42 nt

Other used oligonucleotides

Oligo no.	Name	Sequence (5'-->3')	length
KD035	FAM-IABkFQ reporter	/56-FAM/TTATT/3IABkFQ/	5 nt

Supplementary Table S2. Plate reader settings. Related to STAR Methods – Method details: Fluorescent reporter trans-cleavage assays. For kinetic fluorescence monitoring, we used the following settings on the Infinite® 200 Pro M Plex plate reader (Tecan Group Ltd.).

**Mode**	**Fluorescence top reading**
Temperature	37 °C
Excitation	485 nm
Emission	535 nm
Excitation bandwidth	9 nm
Emission bandwidth	20 nm
Gain	85 (manual)
Number of flashes	10
Integration time	20 µs
Lag time	0 µs
Settle time	0 µs
Z-position (manual)	16814 µM
